# Predictive Modeling of Proteins Encoded by a Plant Virus Sheds a New Light on Their Structure and Inherent Multifunctionality

**DOI:** 10.3390/biom14010062

**Published:** 2024-01-02

**Authors:** Brandon G. Roy, Jiyeong Choi, Marc F. Fuchs

**Affiliations:** Plant Pathology and Plant-Microbe Biology Section, School of Integrative Plant Science, Cornell University, 15 Castle Creek Drive, Geneva, NY 14456, USA; jc3398@cornell.edu (J.C.); marc.fuchs@cornell.edu (M.F.F.)

**Keywords:** virus, modeling, protein, prediction, function, in silico, nepovirus, AlphaFold2

## Abstract

Plant virus genomes encode proteins that are involved in replication, encapsidation, cell-to-cell, and long-distance movement, avoidance of host detection, counter-defense, and transmission from host to host, among other functions. Even though the multifunctionality of plant viral proteins is well documented, contemporary functional repertoires of individual proteins are incomplete. However, these can be enhanced by modeling tools. Here, predictive modeling of proteins encoded by the two genomic RNAs, i.e., RNA1 and RNA2, of grapevine fanleaf virus (GFLV) and their satellite RNAs by a suite of protein prediction software confirmed not only previously validated functions (suppressor of RNA silencing [VSR], viral genome-linked protein [VPg], protease [Pro], symptom determinant [Sd], homing protein [HP], movement protein [MP], coat protein [CP], and transmission determinant [Td]) and previously identified putative functions (helicase [Hel] and RNA-dependent RNA polymerase [Pol]), but also predicted novel functions with varying levels of confidence. These include a T3/T7-like RNA polymerase domain for protein 1A^VSR^, a short-chain reductase for protein 1B^Hel/VSR^, a parathyroid hormone family domain for protein 1E^Pol/Sd^, overlapping domains of unknown function and an ABC transporter domain for protein 2B^MP^, and DNA topoisomerase domains, transcription factor FBXO25 domain, or DNA Pol subunit cdc27 domain for the satellite RNA protein. Structural predictions for proteins 2A^HP/Sd^, 2B^MP^, and 3A^?^ had low confidence, while predictions for proteins 1A^VSR^, 1B^Hel*/VSR^, 1C^VPg^, 1D^Pro^, 1E^Pol*/Sd^, and 2C^CP/Td^ retained higher confidence in at least one prediction. This research provided new insights into the structure and functions of GFLV proteins and their satellite protein. Future work is needed to validate these findings.

## 1. Introduction

Predictive modeling is increasingly relevant in virus-host interaction research with regard to functional predictions of virus proteins and their interactions with host proteins [1,2,3,4]. The information gained from prediction models can be used to better understand the nature of virus-virus, virus-host, and host-host protein interactions during viral infection. For example, powerful computational methods can predict protein structure and protein functions from viral amino acid sequences [2,3,4]. In cases where the structure of a similar protein to a candidate viral protein is experimentally determined, algorithms based on modeling can provide accurate predictions of the protein structure. When no sequences of similar proteins to the candidate viral protein are available, AlphaFold, a deep learning system, is widely utilized to predict the three-dimensional protein structure by “free modeling” [3].

Grapevine fanleaf virus (GFLV) causes fanleaf degeneration, one of the most devastating viral diseases of grapevine (*Vitis* spp.) in most vineyards worldwide [5,6,7]. This disease was first described in the late 1800’s [8], but it was not until the early 1960’s that GFLV was identified as a causative agent [9]. GFLV is transmitted by the ectoparasitic dagger nematode, *Xiphinema index*, in a semi-persistent manner [10,11]. Infections of GFLV can reduce crop yield by up to 80% and elicit a progressive decline of vines that limits the productive lifespan of a vineyard [5].

**Figure 1:**
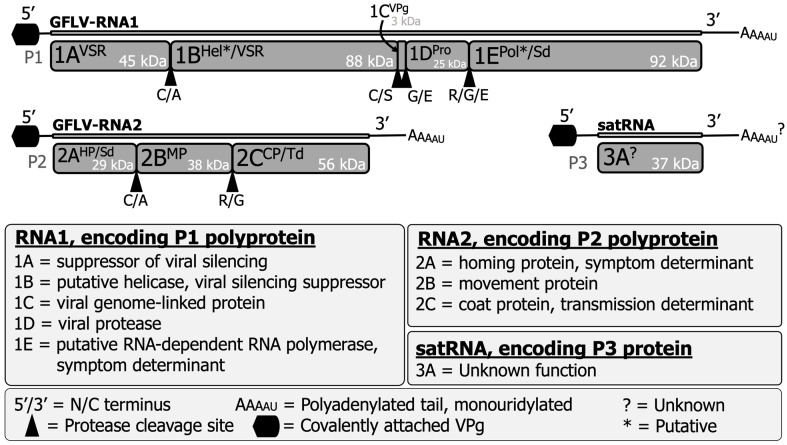
Structure and expression of the grapevine fanleaf virus genomic RNA1, RNA2, and satRNA. These molecules encode five, three, and one mature protein(s), respectively. Associated and biologically validated functions are shown in the legend, along with putative functions denoted by asterisks (*) and unknown functions or unknown sequences are denoted by question marks (?). Proteolytic cleavage sites are indicated by solid triangles. The molecular weight for each protein (kDa) was updated using current bioinformatic predictions via The Sequence Manipulation Suite [13].

GFLV is a member of the species *Nepovirus foliumflabelli* of the family *Secoviridae* [12]. The genome of GFLV is bipartite and consists of two positive-sense, single-stranded RNAs: RNA1 and RNA2 (Figure 1) [12,13]. GFLV RNA1 can replicate solely in single plant cells, but both genomic RNAs are required for systemic plant host infection [14]. The GFLV genome is encapsidated into icosahedral particles with a pseudo *T* = 3 symmetry and a diameter of approximately 28 nm [15,16]. The 5′ end of the genomic RNAs is attached to a viral genome-linked protein (VPg, 1C^VPg^), and the 3′ end is polyadenylated and mono-uridylated [16,17]. GFLV RNA1 (7342 nts) and RNA2 (3806 nts) encode polyprotein P1 (253 kDa) and polyprotein P2 (123 kDa), respectively [5,15,16].

GFLV P1 and P2 are proteolytically cleaved by the RNA1-encoded 3C-like protease (Pro; 1D^Pro^) upon monocistronic translation [18]. GFLV P1 is processed in *cis*, resulting in five individual, mature proteins: protein 1A^VSR^ (45 kDa), a viral silencing suppressor (VSR); protein 1B^Hel*/VSR^ (88 kDa), a putative helicase (Hel*) and a VSR; protein 1C^VPg^ (3 kDa), a VPg; protein 1D^Pro^ (25 kDa), a proteinase; and protein 1E^Pol*/Sd^ (92 kDa), a putative RNA-dependent RNA polymerase (RdRP*) and a symptom determinant in the model herbaceous host *Nicotiana benthamiana* (Figure 1) [5,16,19,20,21,22]. GFLV P2 is processed in *trans*, resulting in three mature proteins: protein 2A^HP/Sd^ (29 kDa), a homing protein (HP) that directs RNA2 to the virus replication sites [23] and elicits a hypersensitive reaction on *N. occidentalis* [24]; protein 2B^MP^ (38 kDa), a movement protein (MP) that facilitates cell-to-cell movement of virions via plasmodesmata in a tubule-guided manner [5,25,26]; and protein 2C^CP/Td^ (56 kDa), the structural coat protein (CP) that composes virions and determines nematode transmission specificity (Figure 1) [16,27,28,29,30]. The crystal structure of protein 2C^CP/Td^ was determined at a 2.7 Å resolution [27,28,29,30]. The function(s) of GFLV-encoded proteins were predicted based on conserved, canonical amino acid sequences or confirmed through biological validation assays.

Some GFLV isolates contain an extraneous RNA molecule referred to as a satellite (sat) RNA or RNA3 (Figure 1) [31,32,33]. The size of the nongenomic GFLV satRNA varies from 1,104 to 1,140 nts and encodes a single nonstructural protein 3A^?^ (37 kDa), which is also referred to as protein P3 [31,32,33,34]. GFLV satRNA molecules belong to two phylogenetic clades based on their nucleotide or amino acid sequence variability [31]. GFLV satRNA replication and encapsidation depend on its helper virus. The GFLV satRNA does not affect virus accumulation or symptom expression either in the natural host, grapevine [34], or in the experimental model herbaceous host, *Chenopodium quinoa* [35]. The function(s) of protein 3A^?^ has yet to be elucidated. 

Despite substantial advances in GFLV biology, many aspects of GFLV-encoded protein structure and function, as well as their potential roles in molecular and cellular processes that govern virus-host interactions, have not been elucidated. We hypothesized that publicly available, open source bioinformatic tools and computational modeling software would not only confirm previous findings regarding GFLV protein function and structure but also provide new insights into viral protein domain function beyond a simple analysis of primary sequence composition. The combination of previously performed functional assays and localization studies, the availability of a protein 2C^CP/Td^ crystal structure, and the identification of many putative functions make this plant virus a great case study for leveraging functional protein predictive modeling. Here, we present our findings on the predictive modeling of GFLV proteins, analyze our data considering current understandings in the literature, and suggest future biological studies.

## 2. Materials and Methods

### 2.1. GFLV Sequence Retrieval and Curation

GFLV strains F13 and GHu were selected for this study for their extensive biological characterization and genetic distance [30,32,36,37,38]. Amino acid sequences of GFLV proteins encoded by RNA1, RNA2, and satRNA were retrieved from NCBI entries (Table S1). Sequences of GFLV RNA1, RNA2, and satRNA were manually parsed for the nine encoding regions in Jalview [v.2.11.2.6] (Tables S1 and S2) [39].

### 2.2. Disorder and Secondary Structure Prediction

**Figure 2:**
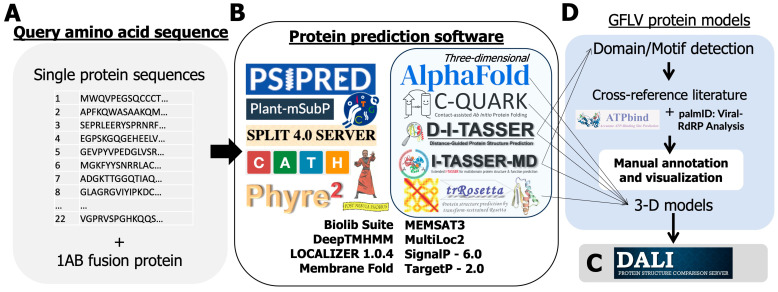
Description of a suite of openly available software used in a protein structure and function prediction pipeline to document the grapevine fanleaf virus (GFLV) protein structure and function. (**A**) A total of 22 GFLV sequences were submitted for analysis in a suite of protein motif and three-dimensional prediction software. (**B**) Some modeling software integrated both folding algorithms with follow-up functional analysis, (**C**) Other software performed one task, (**D**) and All programs were compared and curated to generate a consensus prediction of function(s). Programs are under CC BY 4.0 License with the ability to share with proper citation, as in the methods.

Secondary structure prediction was determined intrinsically in 3D pipelines (Figure 2) or was produced by PyMOL v2.5.1 (Schrodinger, LLC, New York, NY, USA) upon visualization.

### 2.3. In Silico Protein Modeling

The amino acid sequences of GFLV proteins were subjected to several 3D protein modeling servers for in silico predictions. Most GFLV proteins are non-homologous to known structures apart from protein 2C^CP/Td^, for which the structure was determined at a 2.7 Å resolution [27,28,29,30,40]. Therefore, all varieties of protein structure prediction tools, ranging from ab initio to homology-guided software, were selected and utilized (Figure 2). First, GFLV protein amino acid sequences were submitted to AlphaFold2, specifically ColabFold v1.5.2, with default parameters to construct simple 3D models [41,42,43]. Then, GFLV protein amino acid sequences were submitted to the Deep-learning based Iterative Threading ASSEmbly Refinement (D-I-TASSER) server [44,45,46,47] to reconstruct AlphaFold2 predictions and produce structure-based protein function annotations via BioLiP [48]. Any single protein sequence that is too large for D-I-TASSER was alternatively analyzed by Extended I-TASSER for multidomain protein structure and function predictions (I-TASSER-MTD) [49,50]. Any protein sequence that retained a TM-score of less than 0.50 by both algorithms was then subject to Contact-guided Iterative Threading ASSEmbly Refinement (C-I-TASSER), an expansion of I-TASSER that uses inter-residue contact maps and neural net prediction capabilities [51]. For further 3D motif detection, the highest-ranking pdb file generated for each protein was submitted to DALI for structural analysis [52,53]. The highest TM-scoring prediction of the I-TASSER suite was used for the main functional prediction to retain consistency through COFACTOR [54,55].

Sequences were also submitted to ESMFold [56], RGN2 [57], and ProtGPT2 [58], which are designed as single-sequence or non-homologous protein prediction language models. De novo structure prediction was performed by trRosetta [59,60]. Ab initio structure prediction was performed with Contact-assisted QUARK (C-QUARK) [61]. A combined ab initio and comparative folding pipeline called Robetta (https://robetta.bakerlab.org/, accessed on 6 September 2023) [62,63,64] which combines ROSETTA use of protein folding with HHSEARCH [65,66], SPARKS-X [67], and RaptorX [68] programs were utilized. Default parameters were used for sequences submitted to the Robetta server and recorded according to top model predictions for each protein submitted.

### 2.4. Domain, Motif, and Structural Site Predictive Modeling

The GFLV protein sequences and 3D models were utilized in predictive modeling software to identify putative functions. As previously described, BioLiP is integrated into the D-I-TASSER pipeline to generate gene ontology (GO) predictions based on molecular function, biological process, and cellular compartment [48]. Additional motif detection was performed against the five proteins encoded by GFLV RNA1 (1A^VSR^, 1B^Hel*/VSR^, 1C^VPg^, 1D^Pro^, 1E^Pol*/Sd^) and three proteins encoded by GFLV RNA2 (2A^HP/Sd^, 2B^MP^, and 2C^CP/Td^) using MOTIF Search (GenomeNet, Kyoto University Bioinformatics Center, Kyoto, Japan) against publicly available PROSITE, NCBI-CDD, and Pfam databases with E-value parameter relaxed to 5.0 [69,70,71,72]. Five programs were used to predict the cellular localization of GFLV proteins. LOCALIZER (v1.0.4) prediction of subcellular localization was utilized on all protein sequences [73]. Plant mSubP [74], MultiLoc2 [75], and TargetP [76] were used as additional algorithms to predict the subcellular localization of GFLV proteins. SignalP was used to detect signal peptide encoding regions on GFLV proteins [77]. The ScanProsite tool (www.prosite.expasy.org/scanprosite/, accessed on 21 June 2023) was used against all proteins with the following modifications to default settings: deselection of the exclusion of motifs with a high probability of occurrence from the scan and selection ‘Run the scan at high sensitivity’ (SIB—Swiss Institute of Bioinformatics) [78]. Certain prediction servers had additional limitations that were not conducive to the prediction of larger proteins of GFLV and were, therefore, excluded from the analysis and methodology.

Analysis of GFLV protein sequences was also performed with the Protein Homology/Analogy Recognition Engine (Phyre2) V2.0 server [79]. The palmID: Viral-RdRP Analysis server (https://www.serratus.io/palmid, accessed on 22 June 2023) was used for protein 1E^Pol*/Sd^ analysis [80]. Functional family annotation was performed using the Cath/Gene3D v4.3 server by submitting GFLV RNA1 and RNA2 amino acid sequences [81,82]. Advanced curation of ATP binding and all nucleotide binding was determined through 3D and 2D methods using ATPbind [83] and NsitePred [84], respectively. The predicted nucleotide binding sites were determined based on the overlap between NsitePred and ATPbind results. Transmembrane domain and membrane helix predictions were analyzed using MembraneFold [85], DeepTMHMM [86], Split 4.0 [87], Phobius [88], MEMSAT3 with PSIPRED [89], and Phyre2 [79]. The predicted transmembrane domains/helices were determined based on the overlap from a minimum of two programs listed.

### 2.5. Statistical Considerations, Graphic Generation, and Visualization

All metrics provided by each software were extracted, as previously described [90,91]. PDB files were visualized in PyMOL v2.5.1 with manual annotation and color schemes. Further, the PyMOL native ‘align’ function was used to assess the predictions of 2C^CP/Td^, whose crystallography structure was previously reported (PDB:5FOJ) [29]. The output of RMSD was extracted and compiled for future analyses of protein structure. Images of protein models and graphics were annotated in Microsoft^®^ PowerPoint (©Microsoft 2023) and Adobe Illustrator^®^ (Adobe Inc., San Jose, CA, USA). Consensus motifs, domains, and functional properties were calculated to scale per sequence in 2D and 3D space unless otherwise indicated. Any proteins without pLDDT metrics were submitted to QMEANDisCo to obtain a confidence metric [91,92].

## 3. Results

### 3.1. Current Documented Features of GFLV Proteins

Putative and experimentally validated functions and cellular localization are known for the eight proteins encoded by GFLV RNA1 and RNA2, and the satRNA (Table 1). Putative functions have been assigned to GFLV proteins 1B^Hel*/VSR^ and 1E^Pol*/Sd^ based on conserved amino acid sequence motifs or similarity, yet experimental confirmation is lacking. 

Among the proteins of GFLV P1, proteins 1A^VSR^ and 1B^Hel*/VSR^ are experimentally confirmed as viral silencing suppressors (VSRs) with the ability to reverse systemic RNA silencing individually or as a fused form (1A^VSR^B^Hel*/VSR^), a predicted intermediary product of proteolytic processing [19]. Protein 1C^VPg^ has been characterized via sequencing [93], and protein 1D^Pro^ cleaves P1 in *cis* and P2 in *trans* through a triad proteolytic pocket [94,95,96,97]. Protein 1E^Pol*/Sd^ is a symptom determinant in *N*. *benthamiana* with vein-clearing symptomology depending on the identity of a single amino residue at amino acid (aa) position 802 [20,21,98,99]. Protein 1B^Hel*/VSR^ localizes in the endoplasmic reticulum [17,88] and is predicted as a putative helicase based on helicase domain homology with picornaviruses [36,38], and protein 1E^Pol*/Sd^ is predicted as a putative RdRP based on conserved sequence homology of RdRP domains in other nepoviruses and picornaviruses [14,36,38]. However, the helicase activity of protein 1B^Hel*/VSR^ and the polymerase function of protein 1E^Pol*/Sd^ are yet to be confirmed experimentally (Figure 1 and Table 1).

Amongst the proteins of GFLV P2, protein 2A^HP/Sd^ is required for RNA2 replication and guides RNA2 to the replication site [23,99]. Protein 2A^HP/Sd^ is found in aggregated forms in the juxtanuclear space, where it colocalizes with proteins 1C^VPg^ and 1D^Pro^ in protoplasts [23]. Protein 2A^HP/Sd^ is also a symptom determinant that triggers a hypersensitive reaction in *N*. *occidentalis* [24]. Protein 2B^MP^ is responsible for tubule-mediated cell-to-cell movement of the virus through plasmodesmata [25,26,99,100]. Protein 2B^MP^ interacts with plasmodesmata located proteins for tubule formation in a myosin-dependent manner [99,100]. Protein 2C^CP/Td^ is composed of three jelly-roll ß-barrel domains called A, B, and C and forms virions [29,30,101]. Protein 2C^CP/Td^ determines the transmission specificity of GFLV by the ectoparasitic dagger nematode *X. index* [27,28,29,30] (Figure 1 and Table 1). It seems that the surface charge of the B domain pocket or ligand-binding pocket dictates nematode vector specificity [28,29].

**Table 1:** List of putative and experimentally confirmed functions and cellular localization of grapevine fanleaf virus (GFLV) encoded proteins and satRNA protein.

GFLV RNA	Protein ^a^	Experimentally Validated Function ^b^	Putative Function ^c^	Confirmed Localization	Reference(s)
RNA1	1A^VSR^	Viral silencing suppressor	^-^	-	[19]
	1B^Hel*/VSR^	Viral silencing suppressor	Helicase	Endoplasmic reticulum	[14,16,19,36,38,102]
	1C^VPg^	Viral genome-linked protein	-	-	[93]
	1D^Pro^	Viral protease	-	-	[22,94,95,96,97]
	1E^Pol*/Sd^	Symptom determinant	RNA-dependent RNA polymerase	-	[20,21,36,38,98,103]
RNA2	2A^HP/Sd^	Homing protein Symptom determinant	-	Perinuclear space	[23,24,99]
	2B^MP^	Movement protein	-	Plasmodesmata	[25,26,99,100]
	2C^CP/Td^	Coat protein Transmission determinant	-	-	[27,28,29,30,95,101]
satRNA	3A^?^	-	-	-	[31,32,33,34,35]

^a^ GFLV proteins with asterisks (*) represent putative functions. ^b^ Function(s) of GFLV proteins experimentally confirmed. ^c^ Putative functions of GFLV proteins predicted based on sequence homology.

The function of the satRNA-encoded protein P3 is unknown [31,32,33,34,35]. No functional predictions have been made on P3 due to its low sequence homology with well-characterized proteins for which sequence information is available in databases (Figure 1 and Table 1).

### 3.2. Predictions of Functions and Structures for GFLV Proteins

**Figure 3:**
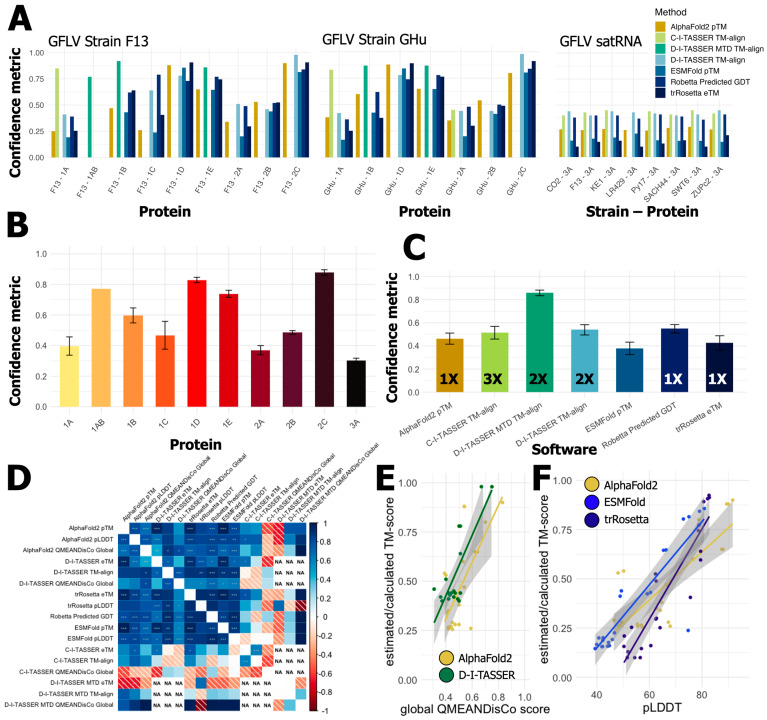
The performance and relative confidence for protein prediction software across the grapevine fanleaf virus (GFLV) proteins revealed trends behind protein-specific and software-specific challenges in producing confident models. (**A**) All protein predictions are shown per program as individual bar graphs separated for GFLV strain F13, GFLV strain GHu, and all 3A^?^ satRNA proteins. The Y-axis shows the confidence metric, while the X-axis shows individual GFLV proteins. (**B**) The average and standard error of protein structure predictions for each individual protein of the GFLV genome and satRNA. (**C**) Average confidence and standard error per software used in the generation of three-dimensional structure prediction for GFLV proteins. Numbers within bars indicate the number of times each software was able to produce the highest confidence prediction per protein. (**D**) Pearson’s correlation matrix shows significant, positive agreement amongst most protein prediction algorithm confidence metrics (*p*-value; * = <0.05, ** = <0.01, *** = <0.001). (**E**,**F**) Linear models of confidence metrics for multiple programs show minor differences on a protein-by-protein basis. (**E**) AlphaFold2 and D-I-TASSER report similar estimated TM-score and global QMEANDisCo scores for each protein prediction. In contrast, (**F**) AlphaFold2 outperforms ab initio programs ESMFold or trRosetta for nearly all GFLV proteins for both estimated TM-scores and pLDDT metrics. (AlphaFold2 = pTM [yellow/brown]; C-I-TASSER = TM-aligned [lime green]; D-I-TASSER-MTD = TM-aligned [dark green]; D-I-TASSER = TM-aligned [light blue]; ESMFold = pTM [blue]; Robetta = predicted GDT [dark blue]; trRosetta = eTM [black]).

Since studies on the functions of GFLV proteins were primarily centered on GFLV strains F13 or GHu, both viral strains were included in our study. Manually curated amino acid sequences for GFLV RNA1 and RNA2 proteins (strains F13 and GHu) and satRNA protein (strains F13, CO2, LR4/29, Py17, SACH44, and SWT6) were analyzed for motif detection and tertiary protein modeling (Table S2 and File S1), and the relative confidence of each modeling output was assessed (Figure 3 and Table S3). These sequences were subject to analyses with the aforementioned 28 bioinformatic programs to probe their structural and functional characteristics (Table 2). The total predicted functions for GFLV strains F13 and GHu are summarized (Table S4) while those with predictions with C-scores of ≥0.30 for GFLV strain F13 (Table 3) and GFLV strain GHu (Table 4) were compiled and tabulated. 

Results revealed that primary sequence alignment software outperformed tertiary protein model predictions in all instances (Figure 3). However, alignment coverage and known sequences may bias this result, which is further confounded by the disparity of known crystallized proteins available for comparison to the predictions. The differences in these types of programs suggested that we separate our analyses into two groupings when attempting to compare protein prediction software, one for motif and specific domain descriptions and the other for overall structural or functional characteristics (File S1). Output of tertiary protein structure amongst available programs can vary in their confidence metric and the intrinsic calculation; however, most are highly correlated [104]. Calculations of eTM/pTM/TM-scores or GDT scores are ranked from 0 to 1, with 1 being of highest confidence (Table 2). Global error scores such as pLDDT or QMEANDisCo are calculated per residue and then averaged on a scale of 0 to 100 or 0 to 1, respectively (Table 2). Most of these metrics consider root mean square deviation (RMSD) error at some stage or are derived directly as RMSD with minor adjustments for length bias or torsion angles. Examination and comparison were conducted with caution for each program against others, on a per protein basis, because not all algorithms provide the same output in the same metrics (Table 2).

In terms of 3-D protein prediction, software performance to confidently predict structure from the primary sequence was highly variable across proteins but was relatively similar across all software. Similar to the performance of AlphaFold2, we found the C-I-TASSER, D-I-TASSER, and D-I-TASSER MTD to be the most beneficial and confident software based on TM-score and comprehensive analysis despite using each in a different instance, (Figure 3 and Table S3). The highest TM-score for proteins solely submitted to the D-I-TASSER server was indeed achieved by the D-I-TASSER server, while the Robetta GDT was of similar confidence (Figure 3 and Table S3). Per the protein 3A^?^ sequences, C-I-TASSER performed just above D-I-TASSER with TM-align scores of 0.426 and 0.424, respectively (Table S3). Pearson’s correlation of all metrics generated from 3D models showed positive, significant associations between protein models (Figure 3D). Variability was observed with the best program on a protein-by-protein basis. Mainly, homology-guided protein prediction models were of similar confidence even if calculated in different ways. Incomplete predictions from D-I-TASSER MTD, mainly where predictions did not need to be performed, left ‘NA’ in the plot (Figure 3D). As with other studies [53,90,91,92], we found significant correlations of model metrics per algorithm. Interestingly, these metrics were not all statistically correlated across algorithms, leaving additional difficulty when discerning the true best model created by each algorithm.

Indeed, AlphaFold2 was able to produce models with minimized conflicting atomic structure (Figure 3E) when compared to D-I-TASSER models. However, pTM/eTM scores indicate that D-I-TASSER was more suitable for protein prediction. It is increasingly obvious that, for ESMFold and trRosetta, the homology-guided AlphaFold2 outperformed every protein of GFLV in terms of pLDDT and predicted TM-scores (Figure 3F). Nonetheless, with this small subset of proteins, it is difficult to discern the ‘best’ prediction method, as positive RNA viruses retain little crystallized entries in the PDB. A unified metric that all programs must provide would be an excellent addition for efficient comparison-making.

Nevertheless, based on the predicted accuracy of each protein encoded by RNA1 (1A^VSR^, 1B^Hel*/VSR^, 1C^VPg^, 1D^Pro^, and 1E^Pol*/Sd^) and two proteins of RNA2 (2A^HP/Sd^ and 2B^MP^), the top model, using the derived TM-score, was deposited into ModelArchive (SIB, Swiss Institute of Bioinformatics, modelarchive.org, accessed on 12 November 2023) under the following identifiers: ma-nqabp, ma-gzevc, ma-3h6c, ma-wg6ry, ma-pufyr, ma-gs2nn, and ma-yhs0u, respectively. We did not deposit any predictive models for protein 2C^CP/Td^ as they were redundant for the already crystallized structure of this protein (PDB 5FOJ) [28,29].

**Figure 4:**
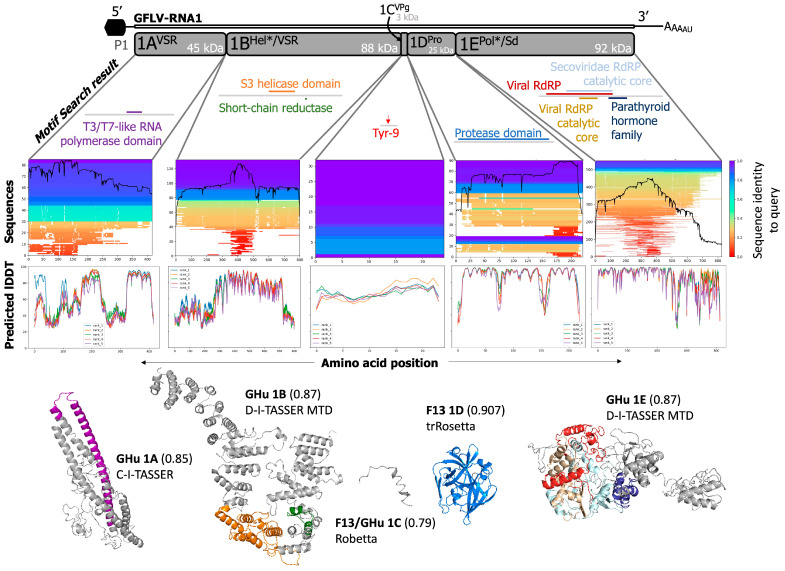
Computational analysis and protein structure prediction of the five mature proteins encoded by grapevine fanleaf virus (GFLV) RNA1. Domain detection returned eight confident predictions of T3/T7-like RNA polymerase domain (protein 1A^VSR^, purple), S3 helicase domain (protein 1B^Hel*/VSR^, orange), short-chain reductase domain (protein 1B^Hel*/VSR^, green), protease domain (protein 1D^Pro^, blue), three RNA-dependent RNA polymerase profiles (protein 1E^Pol*/Sd^, red, light blue, and gold), and parathyroid hormone family domain (protein 1E^Pol*/Sd^, dark blue). The sequence alignment in AlphaFold2 varied from ~30 alignments to over 500 alignments with varying confidence levels based on similarity and coverage against GFLV encoding proteins. Similarly aligned and confident protein sequences returned confident pLDDT upon tertiary protein model generation through five iterations in AlphaFold2. Several programs were utilized to provide the most confident structure, which used D-I-TASSER, Robetta, and trRosetta with predictions ranging in TM-align scores from 0.79 to 0.91. Ribbon diagrams display the predicted motifs and domains in the same color as above, while gray areas are not defined.

**Figure 5:**
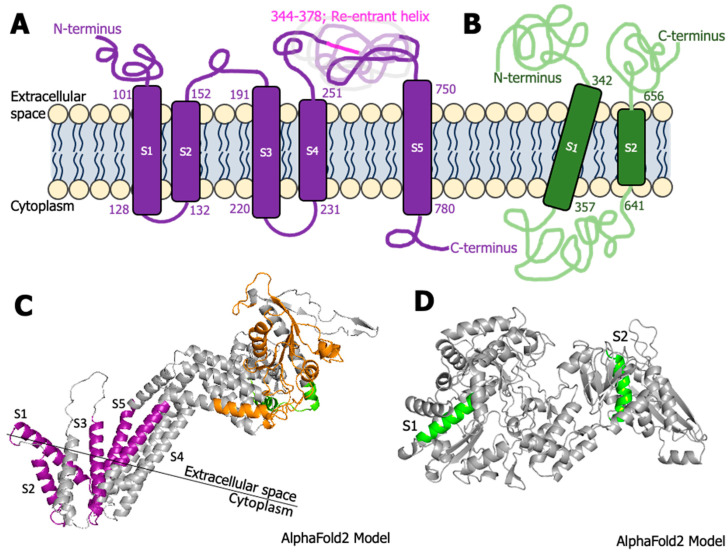
Transmembrane domain predictions by Phyre2 of grapevine fanleaf virus proteins returned only two possible candidates, protein 1B^Hel*/VSR^ and protein 1E^Pol*/Sd^. (**A**) Protein 1B^Hel*/VSR^ was predicted to contain up to five transmembrane domains (purple, transmembrane helices spanning from the extracellular space to the cytoplasm), while (**B**) Protein 1E^Pol*/Sd^ was predicted to contain two transmembrane domains (green). The sequence of amino acids was then projected onto AlphaFold2-generated protein models for visualization. (**C**) The overlap with the sequence of transmembrane domains (purple helices S1–S5) and the predicted protein structure seems likely for protein 1B^Hel*/VSR^ (helicase domain highlighted in orange, reductase in green), but (**D**) additional context is needed on the poorly understood virus replication complex, membrane association predictions (green helices, S1–S2) and behavior of protein 1E^Pol*/Sd^ in planta.

**Figure 6:**
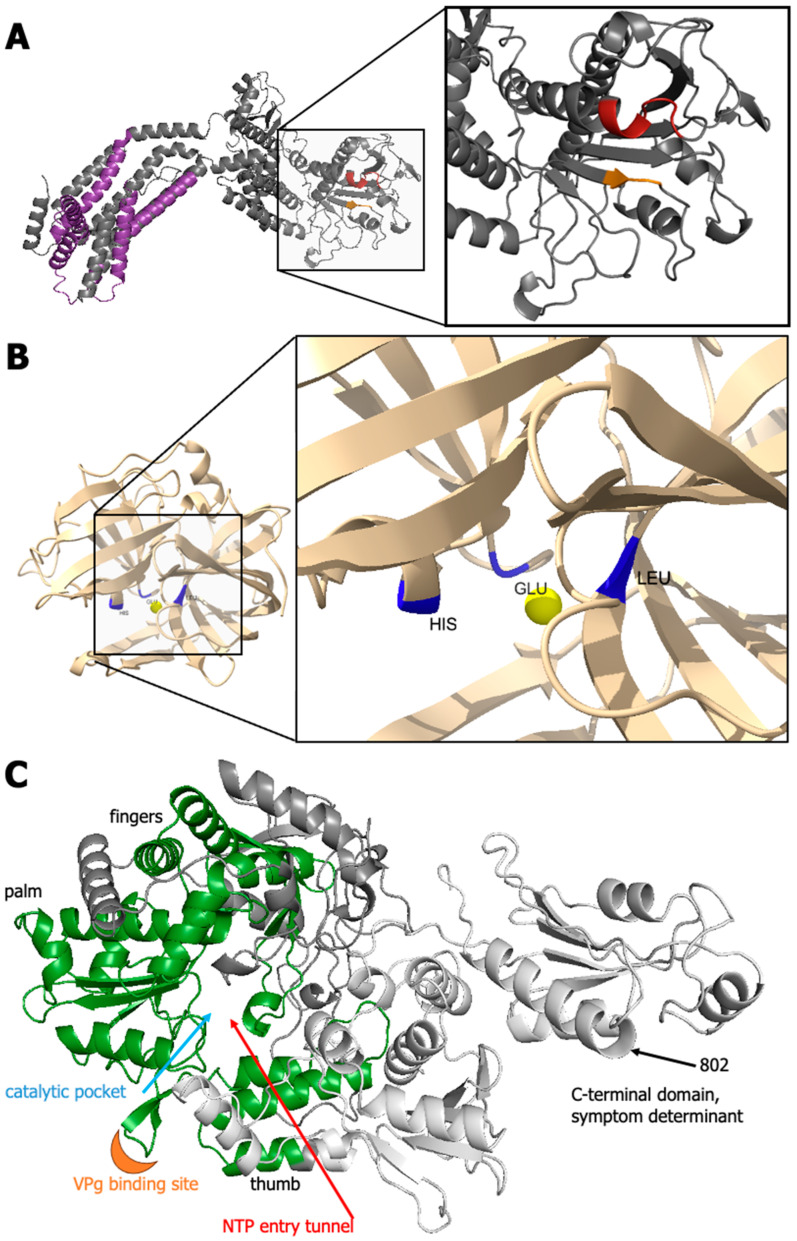
Analyses of the top predictions for proteins of grapevine fanleaf virus (GFLV) to map the putative helicase (1B^Hel*/VSR^), protease (1D^Pro^), and putative RdRP active sites (1E^Pol*/Sd^). (**A**) The AlphaFold2 model of protein 1B^Hel*/VSR^ shows a tertiary structure site for ATP/ADP binding, which aligns with ATPbind and NSitePred predictions at residues 368–373 (in red) and 415–417 (in orange). (**B**) The His-Glu-Leu pocket of protein 1D^Pro^ of GFLV-F13 is highlighted in blue with a putative central proteolysis site indicated by a single yellow sphere (trRosetta model). (**C**) The prediction of protein 1E^Pol*/Sd^ of GFLV-GHu displays consistent structure with other picornaviruses to contain the RdRP palm, fingers, thumb (green), VPg binding site (orange), catalytic pocket, and nucleotide entry site. Residues past 496 of protein 1E^Pol*/Sd^ are colored white as they are not considered part of the polymerase core domain, but rather the C-terminal domain that contains in position 802, the symptom determinant in the herbaceous host *Nicotiana benthamiana*. This image was created with PyMol v2.5.1 (Schrodinger, LLC).

**Figure 7:**
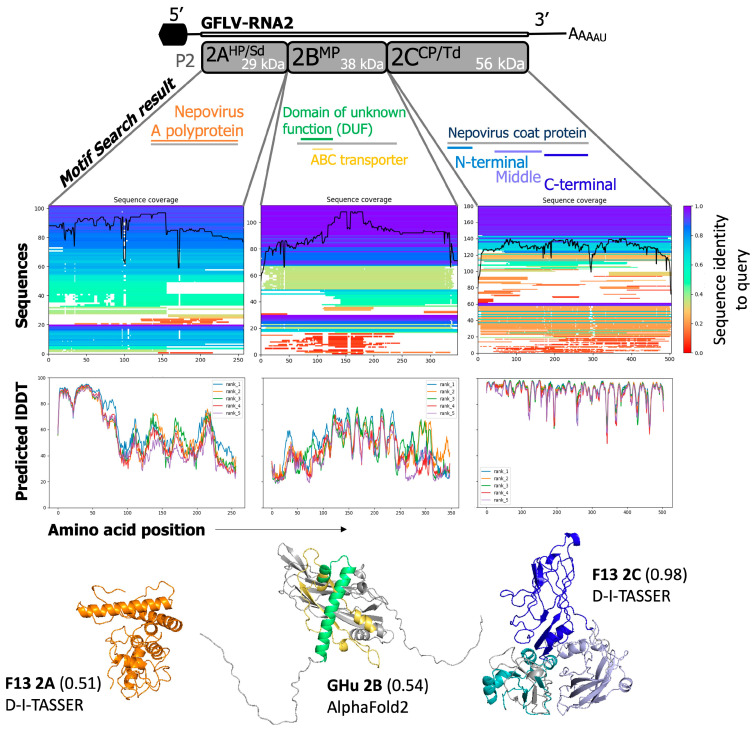
Predicted modeling of the structure and function(s) of proteins encoded by RNA2 of grapevine fanleaf virus (GFLV). The GFLV RNA2 proteins provided confident predictions against the already resolved coat protein structure but less confident and consistent predictions against proteins 2A^HP/Sd^ and protein 2B^MP^. For protein 2A^HP/Sd^, only one motif was detected: nepovirus A polyprotein (orange). This model returned higher for GFLV-F13 using D-I-TASSER with TM-align to result in a TM-score of 0.51. Protein 2B^MP^ returned two consistent motifs of “Domain of unknown function” and “ABC transporter” in green and yellow, respectively. Sequence coverage and identity did not differ greatly from protein 2A^HP/Sd^ and, therefore, resulted in a similar low-confidence model from AlphaFold2 with a pTM-score of 0.54. Protein 2C^CP/Td^ with a crystal structure maintains high sequence similarity to viral coat proteins of nepoviruses. The highest confidence model returned from D-I-TASSER with a TM-score of 0.98. The three coat protein domains A, B, and C are displayed in blue, lavender, and navy on both models.

**Figure 8:**
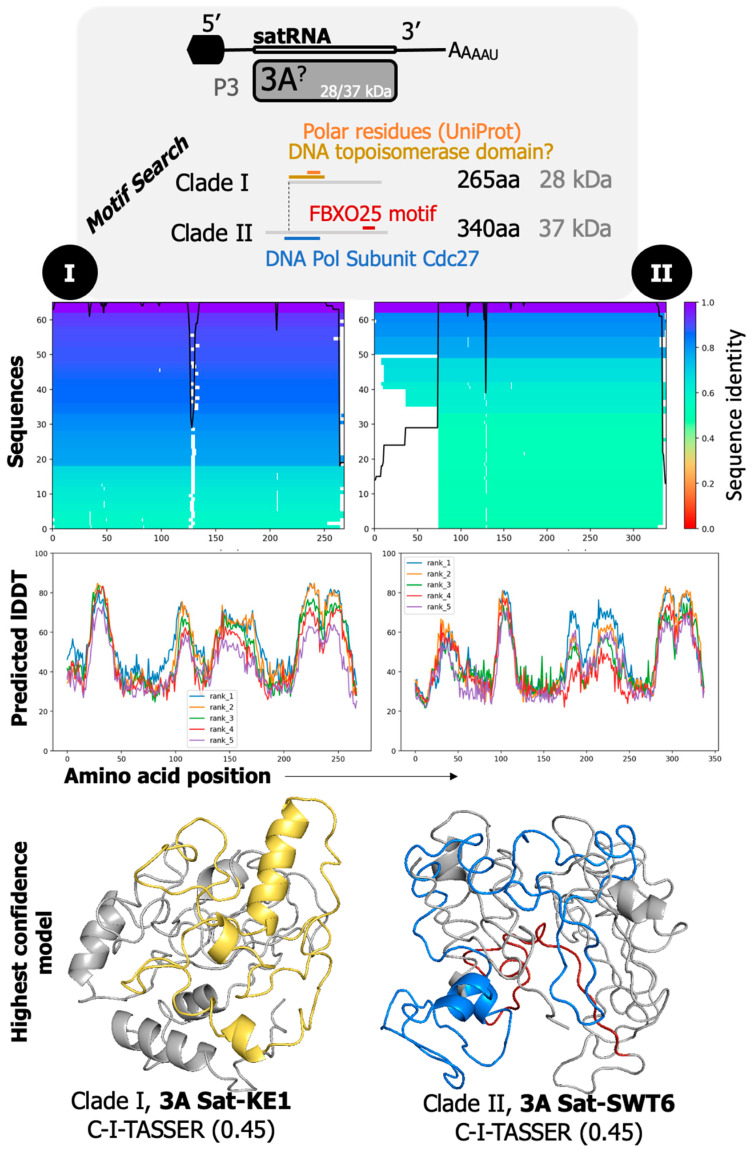
Predicted modeling of the structure and function(s) of the protein encoded by satRNA of grapevine fanleaf virus (GFLV). Protein 3A^?^ of phylogenetic clade I satRNAs (**left**) has a shorter sequence of approximately 265 residues for which GFLV-KE1 returned the highest prediction through C-I-TASSER with a TM-Score through TM-align of 0.45 (yellow pertains to predicted DNA topoisomerase domain). Protein 3A^?^ of phylogenetic clade II satRNAs (**right**) has a longer sequence of approximately 340 amino acids, mostly extended on the N-terminal end when comparing the two sequences, and likewise had poor model generation. Protein 3A^?^ of GFLV-SWT6 returned a TM-score of 0.45 through C-I-TASSER model generation and TM-score alignment (blue and red on 3D model are predicted DNA polymerase subunit cdc27 domain and FBXO25 motif, respectively). While some functions were assigned through Motif Search, their confidence or similarity was low and should be considered with caution.

Results of predictive secondary and tertiary protein structure modeling are presented for each of the eight GFLV encoded proteins, i.e., five proteins in RNA1 and three proteins in RNA2, plus one satRNA protein, as well as the fusion protein 1A^VSR^B^Hel*/VSR^, an intermediary product of polyprotein P1 processing (Figure 4, Figure 5, Figure 6, Figure 7 and Figure 8 and File S1). Image depictions of any generated tertiary structure were the most confident prediction based mostly on TM-score, the most widely accepted metric of protein folding by CASP unless otherwise indicated.

### 3.3. GFLV RNA1 Proteins

Native to the AlphaFold2 Colaboratory resource (https://colab.research.google.com/github/sokrypton/ColabFold/blob/v1.2.0/AlphaFold2.ipynb, accessed on 1 May 2023), MMseqs2 multiple sequence alignments were generated against query sequences of protein-encoding regions of GFLV RNA1 for strains F13 and GHu. 1A^VSR^, 85/96 sequences; 1B^Hel*/VSR^, 132/200 sequences; 1C^VPg^, 32/52 sequences; 1D^Pro^, 90/129 sequences; and 1E^Pol*/Sd^, 550/567 sequences were found at the species/sub-species level (Figure 4). Further, the relative coverage of each of these sequences against the query protein varied for each protein with general trends of linking higher pLDDT of predictions with the most conserved and homologous regions of each protein (Figure 4). All reported molecular function, biological process, and cellular component localization predictions are given by protein and are denoted by a C-score of confidence from 0 (lowest) to 1 (highest) with a gene ontology (GO) identifier (Table 3, Table 4 and Table S4). For additional predictions of the cellular localization of GFLV proteins, predictions from LOCALIZER and those that exhibited a confidence level of <0.70 from four other localization prediction programs were selected in this study (Table S5). The predicted nucleotide binding sites that overlapped between two programs, NsitePred (Table S6) and ATPbind (Table S7), were selected to be presented in this study. Lastly, transmembrane domains/helices that were predicted from a minimum of two out of seven programs were presented (Table S8).

**Table 3:** Output ontologies of proteins encoded by grapevine fanleaf virus (GFLV) strain F13 and satRNA strain F13 from C-I-TASSER, D-I-TASSER, and D-I-TASSER-MTD pipelines. These condensed and parsed results reflect up to three of the nonredundant top ontologies for molecular function, biological process, and cellular components for GFLV proteins.

	Molecular Function			Biological Process			Cellular Component		
GFLV Protein	GO	C-Score ^a^	Name	GO	C-Score	Name	GO	C-Score	Name
1A^VSR^	0015462	0.92	protein-transmembrane transporting ATPase activity	0050658	0.57	RNA transport	0005737	0.94	cytoplasm
	0005524	0.92	ATP binding	0045184	0.57	establishment of protein localization	0043227	0.57	membrane-bounded organelle
							0005634	0.50	nucleus
1B^Hel*/VSR^	0003723	0.50	RNA binding	0039503	0.97	suppression by virus of host innate immune response	0019028	0.85	viral capsid
	0015075	0.49	ion transmembrane transporter activity	0009968	0.96	negative regulation of signal transduction	0033655	0.76	host cell cytoplasm part
	0004386	0.37	helicase activity	0039537	0.95	suppression by virus of host viral-induced cytoplasmic pattern recognition receptor signaling pathway	0033648	0.72	host intracellular membrane-bounded organelle
1C^VPg^	0016798	0.59	hydrolase activity, acting on glycosyl bonds	0044238	0.51	primary metabolic process	0044444	0.67	cytoplasmic part
							0009507	0.33	chloroplast
							0005576	0.33	extracellular region
1D^Pro^	0003824	0.97	catalytic activity	0044003	0.94	modification by symbiont of host morphology or physiology	0016020	0.86	membrane
	0004197	0.78	cysteine-type endopeptidase activity	0039520	0.89	induction by virus of host autophagy			
	0003723	0.71	RNA binding	0039544	0.84	suppression by virus of host RIG-I activity by RIG-I proteolysis			
1E^Pol*/Sd^	0034062	0.68	RNA polymerase activity	0039507	0.97	suppression by virus of host molecular function	0019028	0.85	viral capsid
	0003676	0.63	nucleic acid binding	0039503	0.97	suppression by virus of host innate immune response	0033655	0.76	host cell cytoplasm part
	0035639	0.54	purine ribonucleoside triphosphate binding	0039694	0.77	viral RNA genome replication	0016020	0.63	membrane
2A^HP/Sd^	0046872	0.47	metal ion binding	0044710	0.47	single-organism metabolic process	0016020	0.93	membrane
	0052933	0.37	alcohol dehydrogenase (cytochrome c(L)) activity				0042597	0.64	periplasmic space
							0030288	0.57	outer membrane-bounded periplasmic space
2B^MP^	0046914	0.36	transition metal ion binding	0044710	0.36	single-organism metabolic process	0005576	0.75	extracellular region
							0044464	0.50	cell part
							0031988	0.50	membrane-bounded vesicle
2C^CP/Td^	0005198	0.72	structural molecule activity	0046740	0.65	transport of virus in host, cell to cell	0043231	0.38	intracellular membrane-bounded organelle
							0009341	0.38	beta-galactosidase complex
							0019028	0.31	viral capsid
3A^?^	0046914	0.36	transition metal ion binding	0098662	0.36	inorganic cation transmembrane transport	0016020	0.94	membrane
	0016676	0.36	oxidoreductase activity, acting on a heme group of donors, oxygen as acceptor	0045333	0.36	cellular respiration	0044464	0.89	cell part
	0015078	0.36	hydrogen ion transmembrane transporter activity	0015992	0.36	proton transport	0005886	0.78	plasma membrane

^a^ Each prediction is provided with a C-score ranking, with 1 being the highest confidence and 0 being the lowest confidence.

**Table 4:** Output ontologies of proteins encoded by grapevine fanleaf virus (GFLV) strain GHu from C-I-TASSER, D-I-TASSER, and D-I-TASSER-MTD pipelines. These condensed and parsed results reflect up to three of the nonredundant top ontologies for molecular function, biological process, and cellular components for GFLV proteins.

	Molecular Function			Biological Process			Cellular Component		
GFLV Protein	GO	C-Score ^a^	Name	GO	C-Score	Name	GO	C-Score	Name
1A^VSR^	0097493	0.92	structural molecule activity conferring elasticity	0044763	0.94	single-organism cellular process	0044444	0.97	cytoplasmic part
	005101	0.92	actin filament binding	0045944	0.34	positive regulation of transcription from RNA polymerase II promoter	0044446	0.97	intracellular organelle part
	0005524	0.41	ATP binding	0043123	0.34	positive regulation of I-kappaB kinase/NF-kappaB signaling	00043234	0.94	protein complex
1B^Hel*/VSR^	0003824	0.49	catalytic activity	0080134	0.93	regulation of response to stress	0043231	0.85	intracellular membrane-bounded organelle
	0070182	0.36	DNA polymerase binding	0019054	0.93	modulation by virus of host process	0005634	0.81	nucleus
				0039537	0.92	suppression by virus of host viral-induced cytoplasmic pattern recognition receptor signaling pathway	0044444	0.41	cytoplasmic part
1C^VPg^	0016798	0.59	hydrolase activity, acting on glycosyl bonds	0044238	0.51	primary metabolic process	0044444	0.67	cytoplasmic part
							0009507	0.33	chloroplast
							0005576	0.33	extracellular region
1D^Pro^	0003824	0.98	catalytic activity	0044003	0.94	modification by symbiont of host morphology or physiology	0019028	0.97	viral capsid
	0004197	0.81	cysteine-type endopeptidase activity	0039520	0.89	induction by virus of host autophagy	0016020	0.80	membrane
	0005524	0.74	ATP binding	0039544	0.85	suppression by virus of host RIG-I activity by RIG-I proteolysis			
1E^Pol*/Sd^	0003968	0.63	RNA-directed RNA polymerase activity	0019054	0.99	modulation by virus of host process	0019028	0.87	viral capsid
	0003723	0.52	RNA binding	0039503	0.97	suppression by virus of host innate immune response	0030430	0.76	host cell cytoplasm
	0022838	0.43	substrate-specific channel activity	0039694	0.78	viral RNA genome replication	0033648	0.75	host intracellular membrane-bounded organelle
2A^HP/Sd^	0005088	0.46	Ras guanyl-nucleotide exchange factor activity	0051345	0.56	positive regulation of hydrolase activity	0044424	0.75	intracellular part
				0043087	0.56	regulation of GTPase activity	0016020	0.75	membrane
				0035556	0.56	intracellular signal transduction	0005829	0.62	cytosol
2B^MP^							0005576	0.78	extracellular region
							0043227	0.72	membrane-bounded organelle
							0043234	0.50	protein complex
2C^CP/Td^	0005198	0.72	structural molecule activity	0046740	0.64	transport of virus in host, cell to cell	0043231	0.38	intracellular membrane-bounded organelle
							0009341	0.38	beta-galactosidase complex
							0019028	0.31	viral capsid

^a^ Each prediction is provided with a C-score ranking, with 1 being the highest confidence and 0 being the lowest confidence.

#### 3.3.1. GFLV 1A^VSR^

The functions of protein 1A^VSR^ (416 aa) are unresolved and have low confidence from the absence of similar sequences in protein repositories. Regardless, Motif Search predicted three motifs within the GFLV-F13 sequence, a T3/T7-like RNA polymerase, a STIM1 Orai1-activating region, and a protein of unknown function at aa 195–269, 196–242, 386–403, respectively (Figure 4 and Table S4). The molecular function prediction for protein 1A^VSR^ of GFLV-F13 returned two results of protein-transmembrane transporting ATPase (GO:0015462) and ATP binding (GO:0005524), both with C-scores of 0.92 (Table 3). Interestingly, protein 1A^VSR^ of GFLV-GHu was predicted to have molecular functions such as structural molecule activity conferring elasticity (GO:0097493) and actin filament binding (GO:0051015) with a C-score of 0.92 in addition to ATP binding (GO:0005524) with a C-score of 0.41 (Table 4 and Table S4). The biological process predictions were RNA transport (GO:0050658) and establishment of protein localization (GO:0045184) for protein 1A^VSR^ of GFLV-F13, both with a C-score of 0.57 (Table 3 and Table S4), as well as single-organism cellular process (GO:0044763, C-score = 0.94), positive regulation of transcription from RNA polymerase II promoter (GO:0045944, C-score = 0.34), positive regulation of I-kappaB kinase/NF-kappaB signaling (GO:0043123, C-score = 0.34) for protein 1A^VSR^ of GFLV-GHu (Table 4 and Table S4). No biological process or molecular function was predicted to directly relate to the described VSR function of 1A^VSR^ [19] (Table 3, Table 4 and Table S4). Predictions of cellular localization for protein 1A^VSR^ included cytoplasm (F13, GO:0005737, C-score = 0.94) or cytoplasmic part (GFLV-GHu, GO:0044444, C-score = 0.97), membrane-bounded organelle (F13, GO:0043227, C-score = 0.57), intracellular organelle part (GFLV-GHu, GO:0044446, C-score = 0.97), nucleus (GFLV-F13 and -GHu, GO:0005634, C-score = 0.50), and protein complex (GFLV-GHu, GO:0043234, C-score = 0.94) C (Table 3, Table 4 and Table S4) according to C-I-TASSER, D-I-TASSER, and D-I-TASSER-MTD pipelines, while it was predicted to be localized in the nucleus according to LOCALIZER program (Table S5).

Some of the nucleotide-binding sites based on binding potential to five ligands (ATP, ADP, AMP, GTP, and GDP) were predicted for protein 1A^VSR^ but at low probability < 0.30 and with inconsistent numbers of binding sites between GFLV strains F13 (17 sites) and GHu (seven sites) (Tables S6 and S7). Transmembrane domains or helices were not observed across a minimum of two out of the seven programs that we used (Table S8).

#### 3.3.2. GFLV 1B^Hel*/VSR^

GFLV protein 1B^Hel*/VSR^ (801 aa) had predicted sequences of known and previously undescribed functions. Phyre2 aligned 32% of the amino acid sequence with a prediction confidence of 99.8% (Figure 3 and Table S3). Tertiary de novo modeling returned TM-aligned scores of up to 0.87 against known structures through D-I-TASSER-MTD (Figure 3 and Figure 4 and Table S3). The model with the highest confidence contained many alpha helices stacking throughout a ‘U’ shape, where the two predicted functional motifs lie (Figure 4).

Helicase activity is a previously described putative function of protein 1B^Hel*/VSR^ [14,36], which aligned with Motif Search to be located between residues 361 and 465 for the “superfamily 3 helicase of positive ssRNA viruses” domain profile (Figure 4 and Table S4). An additional motif was predicted as a short-chain dehydrogenases/reductases family signature at residues 564–592 (Figure 4 and Table S4). Molecular functions were predicted as RNA binding activity (GFLV-F13, GO:0003723), ion transmembrane transporter activity (GFLV-F13, GO:0015075), helicase activity (GFLV-F13, GO:0004386), catalytic activity (GFLV-GHu, GO:0003824), and DNA polymerase binding (GFLV-GHu, GO:0070182) with a C-score of 0.5, 0.49, 0.37, 0.49, and 0.36, respectively (Table 3, Table 4 and Table S4).

A total of 25 biological processes were predicted for protein 1B^Hel*/VSR^ of GFLV-F13 and 30 biological processes for that of GFLV-GHu in which 14 of 25 and 13 of 30, respectively, had C-scores above 0.90 (Table S4). Several predicted processes are involved in cellular metabolism including, but not limited to organic substance metabolic process (GFLV-GHu, GO:0071704, C-score = 0.97), primary metabolic process (GFLV-F13 and -GHu, GO:0044238, C-score = 0.98 and 0.97, respectively), macromolecule metabolic process (GFLV-F13 and -GHu, GO:0043170, C-score = 0.98 and 0.96, respectively), multi-organism metabolic process (GFLV-F13 and -GHu, GO:0044033, C-score = 0.97 and 0.93, respectively) (Table S4). Other predictions were related to involvement in host immunity, stress response, or signaling pathways: suppression by virus of host innate immune response (GFLV-F13, GO:0039503, C-score = 0.97), negative regulation of signal transduction (GFLV-F13, GO:0009968, C-score = 0.96), suppression of host viral-induced cytoplasmic pattern recognition receptor signaling pathway (GFLV-F13 and -GHu, GO:0039537, C-score = 0.95 and 0.92, respectively), regulation of response to stress (GFLV-GHu, GO:0080134, C-score = 0.93), and modulation of host process (GFLV-GHu, GO:0019054, C-score = 0.93) (Table 3, Table 4 and Table S4). From the tertiary predictions of D-I-TASSER-MTD, the protein was predicted to be found or associated within the viral capsid (GFLV-F13, GO:0019028, C-score = 0.85), host cell cytoplasm part (GFLV-F13, GO:0033655, C-score = 0.76), or cytoplasmic part (GFLV-GHu, GO:0044444, C-score = 0.41), and host intracellular membrane-bounded organelle (GFLV-F13 and -GHu, GO:0033648, C-score = 0.72 and 0.85, respectively) (Table 3, Table 4 and Table S4). No specific subcellular localization with prediction confidence > 0.60 was observed for protein 1B^Hel*/VSR^ from five localization predicting programs used in this study (Table S5).

Protein 1B^Hel*/VSR^ contained the majority of the predicted nucleotide binding sites with high probability (>0.8) among the GFLV-encoded proteins: S367, Q368, S369, K371, T372 (GFLV-GHu only), and T373 (GFLV-F13 only) or I373 (GFLV-GHu only) (Tables S6 and S7). Residues S367, Q368, and S369 specifically showed a high probability (approximately 1.0) at binding to GDP, while Q368 showed a high probability (≥0.8) at binding to GTP (both GFLV-F13 and -GHu) or GTP and ADP (GFLV-GHu only) (Table S6). Those three residues were also predicted to bind to ATP (Table S7). Phyre2 predicted five intermembrane domains (S1; aa 101–128, S2; aa 132–152, S3; aa 191–220, S4; aa 231–251, and S5; aa 750–780) within protein 1B^Hel/VSR^ of GFLV-F13 (Figure 5A,C). Several transmembrane domains/helices were predicted among four of the seven programs, with three common overlapping domains at positions 102–119, 132–151, and 754–779 for GFLV-F13 and 96–116, 131–151, and 753–776 for GFLV-GHu (Table S8).

#### 3.3.3. Fusion Protein GFLV-1A^VSR^B^Hel*/VSR^

GFLV protein 1A^VSR^B^Hel*/VSR^ is a suppressor of RNA silencing [19]. Results obtained with I-TASSER-MTD showed a TM-score of 0.77 in the determination of gene ontology properties using the MetaGO program against PDB structure 6BFI, a viniculin homolog in a sponge organism [105]. The molecular functions of VIN1 include cytoskeletal protein binding, actin binding, and protein binding [105]. Biological processes, including cell adhesion, cellular process, and cellular component annotations of VIN1, were ubiquitous across the cell [105].

Similar annotations for protein 1A^VSR^B^Hel*/VSR^ through the MetaGO server yielded molecular functions of cytoskeletal protein binding, actin binding, protein complex binding, cell adhesion molecule binding, RNA binding, and eukaryotic initiation factor 4E binding (Table S4 and File S1). Biological processes were more expansive to include broadly cellular process, biological regulation, regulation of cellular process (both positive and negative), regulation of multicellular organismal process, response to stimulus, negative regulation of signal transduction, and single-organism cellular process (Table S4 and File S1).

A TM score of 0.77 was produced by the MetaGO program in the determination of structural homologs, including the top candidate, PDB entry, 82AQ (Table S3). This protein is a DNA-binding protein from the Fanconi anemia group D2, or FANCD2, under the same umbrella of over 22 FANC proteins. This protein is crucial in DNA damage repair mechanisms and carries out several functions of signal transduction, DNA damage response, damage-sensing, and DNA repair [106].

#### 3.3.4. GFLV 1C^VPg^

Given its small size, GFLV protein 1C^VPg^ (24 aa) is not optimal for tertiary prediction programs that rely on iterative overlapping sequences to construct alpha helices and beta sheets for confident scoring structures. The penalties associated with attempts to use the 24 aa long protein resulted in errors for most programs, and Phyre2 is unable to accept such short sequences. Regardless, a tertiary structure was completed by D-I-TASSER with a TM-score of 0.64 with two small alpha helices that minimize folding energy (Figure 3 and Table S3). Robetta created an ab initio form with a GDT of 0.79 (Figure 3 and Figure 4 and Table S3). The final form of the protein may look different in the predicted 5′-RNA complex or in complex with other proteins. Additionally, this protein is identical in amino acid sequence between GFLV strains F13 and GHu (Table S2).

With low confidence (C-score < 0.60), the molecular function of GFLV 1C^VPg^ for both strains F13 and GHu was predicted to be hydrolase activity, acting on glycosyl bonds (GO:0016798), and the biological process was predicted to be primary metabolic process (GO:0044238) (Table 3, Table 4 and Table S4). Scores below 0.60 are not confident and may relate to the restrictions behind iterative homology-guided protein prediction. The cellular component was predicted to be within the cytoplasmic part (GO:0044444, C-score = 0.67) but had other low confidence predictions (C-score < 0.40) in the chloroplast (GO:0009507) and extracellular region (GO:0005576) (Table 3, Table 4 and Table S4). Similar to cellular component results (Table 3 and Table 4), MultiLoc2 predicted cytoplasmic localization for protein 1C^VPg^ with confidence at 0.66 (Table S5).

Nucleotide binding sites could not be predicted for 1C^VPg^ due to its small size (Tables S6 and S7). No transmembrane domains and subcellular localization with high probability (>0.8) were predicted for this protein (Table S8).

#### 3.3.5. GFLV 1D^Pro^

GFLV protein 1D^Pro^ (219 aa) is a highly conserved protease with several closely related proteins with crystal structures. Phyre2 was able to cover 95% of the sequence with 99.5% confidence. Similarly, high levels of confidence were returned with trRosetta de novo modeling with a TM-Score of 0.907 (Figure 3 and Figure 4 and Table S3). D-I-TASSER returned a TM-Score 0.78 (Figure 3 and Table S3). These were extremely high-confidence models compared with those obtained for other GFLV proteins. Many models, including the trRosetta output, contained a single globular domain with beta strands and alpha helices for a tight organization. The proteolytic pocket projected onto the trRosetta prediction indicated the close spatial proximity of residues 43 (His), 87 (Glu), and 197 (Leu) (Figure 6), as previously suggested [97].

With high coverage and alignment, Motif Search predicted the picornaviruses 3C/3C-like protease domain profile (Figure 4 and Table S4), as previously reported [16], to stretch near the entirety of the protein. Additionally, at its N-terminus, the active site was described as coronavirus 6B/7B protein, which is currently undescribed (Figure 4). Eight molecular functions were assigned for this protein for both GFLV strains F13 and GHu, in which the three foremost functions were catalytic activity (GO:0003824, C-score 0.97 and 0.98, respectively), cysteine-type endopeptidase activity (GO:0004197, C-score = 0.78 and 0.81, respectively), and ATP binding (GO:0005524, C-score = 0.71 and 0.74, respectively) (Table 3, Table 4 and Table S4). Eighteen biological processes were assigned, eight of which had C-scores above 0.80: modification by symbiont of host morphology or physiology (GO:0044003), viral process (GO:0016032), primary metabolic process (GO:0044238), protein metabolic process (GO:0019538), induction by the virus of host autophagy (GO:0039520), and suppression by virus of host RIG-I activity by RIG-I proteolysis (GO:0039544) for both GFLV-F13 and -GHu protein 1D^Pro^ (Table 3, Table 4 and Table S4). The cellular localization of the tertiary structure was predicted as membrane-bound (GFLV-F13 and –GHu, GO:0016020, C-score = 0.86 and 0.80, respectively) (Table 3 and Table 4) or viral capsid (GFLV-GHu, GO:0019028, C-score = 0.97) (Table 4). In contrast, MultiLoc2 predicted it to be localized in the cytoplasm with a high probability (≥0.87) (Table S5).

No nucleotide binding sites with a high probability (>0.8) were predicted in our study for protein 1D^Pro^, but it has protein binding sites for its proteolytic cleavage processing [86] (Tables S6 and S7). Despite membrane localization prediction for protein 1D^Pro^ (Table 3 and Table 4), no common transmembrane domains from at least two prediction programs were observed (Table S8).

#### 3.3.6. GFLV 1E^Pol*/Sd^

GFLV protein 1E^Pol*/Sd^ (824 aa) is the largest protein encoded by GFLV. Homology by sequence alignment was highly confident at the N-terminal RdRP domain, in which Phyre2 was able to cover up to 62% of the sequence with 100% confidence (Figure 3 and Table S3). With the overrepresentation of plant virus RdRP sequences in databases, few alignments were made to the C-terminal 328 amino acids that were defined here as the middle and C-terminal domains (Figure 5). Tertiary structure prediction with D-I-TASSER-MTD produced the best TM-score of 0.87, which is very high considering the lack of homologous templates at the C-terminus (Figure 3 and Figure 4 and Table S3). A closer look at homologous templates revealed a heavy indication of coverage towards the N-terminal RdRP and not with the entire length of the protein. A globular structure was mainly found in the polymerase domain. However, the C-terminal end of the protein with little sequence homology contained two smaller domains with increased amounts of disorder (Figure 4 and Figure 6). Domain prediction via Motif Search identified the family *Secoviridae* catalytic core domain of the RdRP (cd23196) (Figure 4 and Table S4).

Molecular function was guided to RdRP activity (GFLV-F13 and -GHu, GO:0034062, C-score = 0.68 and 0.63, respectively), as expected, nucleic acid binding (GFLV-F13, GO:0003676, C-score = 0.63), or RNA binding (GFLV-GHu, GO:0003723, C-score = 0.52), purine ribonucleoside triphosphate binding (GFLV-F13, GO:0035639, C-score = 0.54), and substrate-specific channel activity (GFLV-GHu, GO:0022838, C-score = 0.43) (Table 3, Table 4 and Table S4). For both GFLV strains F13 and GHu, biological processes of suppression of host molecular function (GO:0039507) and host innate immune response (GO:0039503), modulation of host process (GO:0019054) with high confidence were predicted (C-score ≥ 0.97) in addition to viral RNA genome replication (GFLV-F13 and -GHu, GO:0039694, C-score = 0.77 and 0.78, respectively), and suppression by virus of host translation (GFLV-F13 and GHu, GO:0039604, C-score = 0.69 for both) (Table 3, Table 4 and Table S4). Those functions might be related to the symptom-determinant function of 1E^Pol*/Sd^. For protein 1E^Pol*/Sd^ of GFLV strains F13 and GHu, the cellular localization predictions varied from host cytoplasm (Table 3, Table 4 and Table S5) and membrane parts (GO:0033655 and GO:0016020, respectively) to viral capsid (GO:0019028), all of which with C-score above 0.60 (Table 3, Table 4 and Table S4). MuliLoc2 predicted protein 1E^Pol*/Sd^ for both strains GFLV-F13 and –GHu to localize in cytoplasmic component with high confidence (≥0.83) (Table S5).

Protein 1E^Pol*/Sd^ had the most nucleotide binding sites among the GFLV proteins with confidence below 0.80 (Table S6) and a majority (17 to 21 sites) having the binding potential to GDP (17 to 21 sites) and ATP (13 to 15 sites) within positions 197–214 and 276–279, respectively (Tables S6 and S7). Phyre2 predicted two intermembrane domains spanning from the extracellular space into the cytoplasm back into the extracellular space (S1; aa 342–357, S2; aa 641–656) for protein 1E^Pol/Sd^ of GFLV strain GHu (Figure 5B). Interestingly, transmembrane domains in protein 1E^Pol*/Sd^ were observed in both MEMSTAT3 and Phyre2 for GFLV-GHu while observed only in MEMSTAT3 for GFLV-F13 (Table S8). Nonetheless, protein 1E^Pol*/Sd^ for GFLV-GHu was predicted to contain transmembrane domain/helix at positions 342–356 (Table S8).

### 3.4. GFLV RNA2 Proteins

Native to the AlphaFold2 Colaboratory resource (https://colab.research.google.com/github/sokrypton/ColabFold/blob/v1.2.0/AlphaFold2.ipynb, accessed on 1 May 2023), MMseqs2 multiple sequence alignments were generated against query sequences of protein-encoding regions of RNA2 for GFLV strains F13 and GHu. 2A^HP/Sd^, 102/114 sequences; 2B^MP^, 113/122 sequences; and 2C^CP^, 180/154 sequences were found at the species/sub-species level (Figure 7). Again, the relative coverage of each of these sequences against the query protein varied for each protein, with general trends of linking the pLDDT of predictions against the most conserved and similar regions of each protein (Figure 7). All reported molecular function, biological process, and cellular component localization predictions, as well as nucleotide binding sites and transmembrane domains/helices for GFLV RNA2-encoded proteins, are similarly organized as GFLV RNA1-encoded proteins (Table 3, Table 4 and Tables S4–S8).

#### 3.4.1. GFLV 2A^HP/Sd^

GFLV protein 2A^HP/Sd^ (258 aa) is a homing protein and is associated with symptom determination in the model host species *N. occidentalis* [24]. A smaller portion (31%) of the query sequence was covered with high confidence (72%) when GFLV strain GHu was subjected to Phyre2 analysis (Figure 3 and Table S3). Three-dimensional models did not have similar levels of confidence as intracellular proteins are often less abundantly solved for their tertiary structure (Figure 3 and Figure 7 and Table S3). Regardless, the ribbon model generated by D-I-TASSER mainly returned a single globular shape with alpha helices and unordered strands (TM-aligned score of 0.51) (Figure 7).

Domain prediction resulted in the specific annotation towards viral family protein as nepovirus subgroup A polyprotein (PFAM12312) (Figure 7 and Table S4). Specific functional characteristics were described as protein SRG1 (PLN02216) at residues 36–106, which interestingly corresponds to oxidoreductase activity in *Arabidopsis thaliana* and is related to the biological function of leaf senescence (GO:0010150) (Figure 4 and Table S4). Overlapping the report of the 50-distal amino acids of protein 2A^HP/Sd^ involved in symptomatology [24] was the weak prediction of electron transfer flavoprotein subunit alpha (PRK11916, E-value = 2.70). Protein 2A^HP/Sd^ was predicted to have molecular functions of Ras guanyl-nucleotide exchange factor activity (GFLV-GHu, GO:0005088, C-score = 0.46), metal ion binding (GFLV-F13, GO:0046872, C-score = 0.47), alcohol dehydrogenase activity (GFLV-F13, GO:0052933, C-score = 0.37) (Table 3, Table 4 and Table S4). Biological processes related to positive regulation of hydrolase activity (GFLV-GHu, GO:0043087, C-score = 0.56), regulation of GTPase activity (GFLV-GHu, GO:0043087, C-score = 0.56), intracellular signal transduction (GFLV-GHu, GO:0035556, C-score = 0.56), and single-organism metabolic process (GFLV-F13, GO:0044710, C-score = 0.47) were predicted (Table 3, Table 4 and Table S4). The D-I-TASSER models of protein 2A^HP/Sd^ predicted its localization to several parts of the cell, including membrane (GFLV-F13 and GFLV-GHu, GO:0016020, C-score = 0.93 and 0.75, respectively) and periplasmic space (GFLV-F13, GO:0042597, C-score = 0.64) or outer membrane-bounded periplasmic space (GFLV-F13, GO:0030288, C-score = 0.57) (Table 3 and Table 4), intracellular part (GFLV-GHu, GO:0044424, C-score = 0.75), membrane (GFLV-GHu, GO:0016020, C-score = 0.75), and cytosol (GFLV-GHu, GO:0005829, C-score = 0.62) (Table 4) or mitochondria at a high probability (0.76–0.80) (Table S5).

No nucleotide binding sites with high probability (>0.80) were predicted in our study for protein 2A^HP/Sd^ (Table S6), although several ATP binding sites (11 sites for 2A^HP/Sd^ of GFLV-F13 and two sites for 2A^HP/Sd^ of GFLV-GHu) were detected using the ATPbind program (Table S7). No common transmembrane domains/helices for protein 2A^HP/Sd^ were detected from at least two of the seven programs used (Table S8).

#### 3.4.2. GFLV 2B^MP^

GFLV protein 2B^MP^ (348 aa) performed the lowest overall among GFLV proteins in attempts to predict structure and function. Phyre2 was able to cover 40% of the query sequence with up to 7.5% confidence, most of which were of unreliable quality (Figure 3 and Table S3). The estimated internal eTM-Score of D-I-TASSER was better at 0.66, but with TM-aligned validation, it only reached 0.51 (Figure 3 and Table S3). The AlphaFold2 model performed best at 0.54 to contain beta sheets and alpha helices between two arms of disordered random coils (Figure 7).

Domain prediction using Motif Search resolved a motif at residues 66–135 for a periplasmic-binding component of ABC transport systems specific for xylo-oligosaccharides (cd14749), as well as a protein of unknown function motif (DUF448) at residues 25–51 (Figure 7 and Table S4). Molecular function and biological processes returned predictions of low confidence (C-score < 0.30) (Table S4). For protein 2B^MP^ of GFLV-F13, a C-score of 0.36 for transition metal ion binding (GO:0046914) of molecular process and single-organism metabolic process (GO:0044710) of biological process were predicted (Table 3). Protein 2B^MP^ of GFLV-GHu returned low-confidence predictions (C-score < 0.30) for molecular and biological processes (Table S4). Cellular component predictions for protein 2B^MP^ of GFLV-F13 were concentrated to extracellular regions (GO:0005576, C-score = 0.75), membrane-bound vesicle (GO:0031988, C-score: = 0.50), and cell part (GO:0044464, C-score = 0.50) (Table 3 and Table S4). Protein 2B^MP^ of GFLV-GHu retained similar localization predictions, except with the addition of protein complex (GO:0043234, C-score = 0.50) (Table 4). Protein 2B^MP^ was predicted to localize in the cytoplasm (Table S5), consistent with its localization in the plasmodesmata (Table 1) [16,99,100].

Eight to 17 ATP-binding sites were detected for protein 2B^MP^, in which the number of sites varied depending on the program and GFLV strain used (Tables S6 and S7). As observed for protein 2A^HP/Sd^, no consistent transmembrane domain was observed in this study for protein 2B^MP^ (Table S8).

#### 3.4.3. GFLV 2C^CP/Td^

The GFLV protein 2C^CP/Td^ (504 aa) is the only protein of GFLV that has a resolved structure [28,29]. The alignment and tertiary predictions of this molecule retained high confidence at 99% alignment with 100% confidence in Phyre2 and 0.98 TM-align scores through D-I-TASSER (Figure 3 and Table S3). This structure matched the PDB submitted structure, PDB:5FOJ, very closely [28,29].

Domain prediction by Motif Search restated the nepovirus CP by displaying the Pfam signature nepovirus CP N-terminal, central, and C-terminal domains in the following aa regions: 2–91, 166–335, and 344–500, respectively (Figure 7). The alignment of the tertiary shape with true crystal structure was highly accurate regardless of the program and descriptions of function related to already accepted details behind CP subunits (Figure 7). For both GFLV strains F13 and GHu, only one biological process, transport of virus in host cell-to-cell (GO:0046740, C-score ≥ 0.64), was predicted for protein 2C^CP/Td^, along with one molecular function, structural molecule activity (GO:0005198, C-score = 0.72) (Table 3, Table 4 and Table S4). For protein 2C^CP/Td^ of GFLV-F13 and -GHu, the cellular component prediction of D-I-TASSER was split among intracellular membrane-bounded organelle (GO:0043231, C-score = 0.38), beta-galactosidase complex (GO:0009341, C-score = 0.38), and viral capsid (GO:0019028, C-score = 0.31) (Table 3, Table 4 and Table S4). In contrast, no predicted subcellular localization with a high confidence level (>0.8) was detected using LOCALIZER, TargetP, SignalP, Plant mSubP, and MultiLoc2 subcellular localization prediction programs (Table S5).

Several nucleotide binding sites were predicted for protein 2C^CP/Td^, with the majority identified to be ATP or ADP binding sites (five to 10 for ATP and eight to nine for ADP) (Tables S6 and S7). One transmembrane domain was identified for protein 2C^CP/Td^ at positions 239–254 for GFLV-F13 and 238–251 for GFLV-GHu (Table S8).

The alignment of the PDB:5FOJ crystalline structure of protein 2C^CP/Td^ across predicted structures returned with various amounts of confidence (Figure S1), as anticipated. AlphaFold2 predictions of the F13 and GHu strains returned RMSD values of 1.81 and 3.72, respectively. Robetta returned RMSD values over 3.0, and ESMFold returned RMSD values over 1.80. D-I-TASSER had the lowest RMSD values at 1.64 for strain F13 and 1.43 for strain GHu (Figure S1). Prediction algorithms that integrate known homologous structures and multiple sequence alignments, such as D-I-TASSER and AlpahFold2, returned lower error values than *ab initio*-based algorithms.

### 3.5. GFLV satRNA Protein 3A^?^

GFLV satRNA protein 3A^?^ sequences contain a segment of polar residues towards the N-terminus. MotifFinder consistently predicted an F-box domain (F-box domain found in F-box only protein 25 FBXO25) in five protein 3A^?^ sequences towards the C-terminus (Figure 8). Protein 3A^?^ of phylogenetic clade I satRNAs [31] contained a DNA topoisomerase domain at aa 3–100, and that of phylogenetic clade II satRNAs [31] contained two domains, a DNA-polymerase cdc27 subunit at positions aa 46–156 and a F-box domain at positions aa 286–315 (Figure 8). SatRNA protein 3A^?^ of GFLV strain Py17 returned a glutareoxin-like domain with high confidence (E-value = 0.052) (Table S4).

Additionally, protein 3A^?^ of GFLV strains F13, CO2, and SWT6 returned a DNA polymerase subunit cdc27 domain at the N-terminal half (Figure 8 and Table S4). Molecular function, biological process, and cellular component prediction by D-I-TASSER or C-I-TASSER were unreliable, as expected, to span all parts of the cell with no tangential experimental evidence available in the literature. With great amounts of random coils and loops, models were unreliable for protein 3A^?^ (Table S4). Trends began to appear after analyzing all 3A^?^ strain predictions to focus heavily on metal ion binding and catalysis. However, no functional characteristics have been described biologically for satRNA [34,35,36].

Protein 3A^?^ of GFLV satRNA strain F13 was predicted to have several molecular and biological processes at low confidence (C-score < 0.40) which include transition metal ion binding (GO:0046914, C-score = 0.36), oxidoreductase activity (GO:0016676, C-score = 0.36), and hydrogen ion transmembrane transporter activity (GO:0015078, C-score = 0.36), as well as biological processes such as inorganic cation transmembrane transport (GO:0098662, C-score = 0.36), cellular respiration (GO:0045333, C-score = 0.36), and protein transport (GO:0015992, C-score = 0.36) (Table 3 and Table S4). For the cellular localization prediction, protein F13-3A^?^ was predicted to be localized in the membrane (GO:0016020, C-score = 0.94), cell part (GO:0044464, C-score = 0.89), and plasma membrane (GO:0005886, C-score = 0.78) (Table 3 and Table S4) in addition to nucleus or plastid (Table S5).

A total of 9 to 20 nucleotide binding sites were predicted for protein 3A^?^ across different satRNA proteins, yet the probability scores for such predictions were low (<0.50) (Table S6). The ATPbind model was not conducted for 3A^?^ due to low confidence in its protein structure modeling, as mentioned above. The common transmembrane domain from at least two programs was not detected for protein 3A^?^ (Table S8).

## 4. Discussion

Protein structure prediction has blossomed into a beneficial field for guiding new hypotheses, generating short and long-chain three-dimensional structures of protein monomers or multimers [107,108]. The benchmarks of ab initio protein prediction software have highlighted I-TASSER as one of the best current prediction software available [107]. This program and AlphaFold2 were comparable in terms of confident protein structure prediction. However, TASSER programs were increasingly useful for studying biological functions and molecular processes associated with the structure [109]. Initially, we hypothesized that GFLV proteins would contain advanced functions and structures that would deviate from initial training and algorithms of highly rated protein structure and function prediction programs. Our findings revealed that GFLV is still bound to central tenants of protein biology with confident predictions obtained using recent software.

We confirmed previously demonstrated functions for GFLV proteins (Table 1), verified two putative reported functions [14,15,16,36] (Table 1), and documented the predictions of at least seven additional functions through current protein prediction software (Figure 4, Figure 7 and Figure 8 and Table S4). The unresolved crystal structure for seven GFLV proteins, excluding the 2C^CP/Td^ [27,28,29,30], severely limits our understanding of biological functions. We employed the most up-to-date predictive protein modeling tools to gain a new understanding of putative functions and confirm current understandings of functions reported in the literature (Figure 2). The newly predicted functional components were determined to include protein 1A^VSR^ with a T3/T7-like RNA polymerase domain, protein 1B^Hel*/VSR^ with a short-chain reductase, protein 1E^Pol*/Sd^ with a parathyroid hormone family domain, protein 2B^MP^ with overlapping domains of unknown function and an ABC transporter, and protein 3A^?^ to include either DNA topoisomerase domains, the transcription factor FBXO25 domain, or DNA Pol subunit cdc27 domain (Figure 4, Figure 7 and Figure 8 and Table S4). No novel functions were predicted for 1C^VPg^ due to the nature of prediction algorithms and its short aa sequence. The structure and function of 2C^CP/Td^ have been described [27,28,29,30,95,101], and most algorithms were able to predict its structure with ease to include D-I-TASSER, trRosetta, and ESMFold having average RMSD values below 2.0 (Figure S1). AlphaFold2 was not far behind in predicting the structure, with average RMSD values just below 3.0. However, Robetta predictions returned larger RMSD values over 3.0. This instance shows considerable advantages for including template structures in prediction tools when predicting conserved proteins. Overall, our results highlight the importance of incorporating multiple structure prediction algorithms when studying the structure of the protein of interest in silico as the prediction power and error may vary depending on the algorithm (Figure S1). This has been documented in recent reports by CASP [109].

Analyses of GFLV proteins have alluded to the following new predictions (Table 3 and Table 4, Figure 4, Figure 7 and Figure 8 and Table S4) based on structural characteristics. For GFLV RNA1-encoded proteins, protein 1A^VSR^ is involved in transporting activity, cellular reorganization, and regulation of cellular process and signaling; protein 1B^Hel*/VSR^ performs as a helicase, as expected, some form of catalysis, perhaps as a polymerase cofactor [110], while suppressing host immune response and signaling pathways; protein 1C^VPg^ composes hydrolase activity and metabolic process function; protein 1D^Pro^ could interfere with host immune responses through proteolytic activity and/or modify host autophagy events; and protein 1E^Pol*/Sd^ is a suppressor of host immunity, molecular function, and host translation (Table 3, Table 4 and Table S4). These predicted functions for proteins 1A^VSR^, 1B^Hel*/VSR^, and 1E^Pol*/Sd^ might be related to their experimentally confirmed functions as VSRs [19] and symptom determinants [20,21] (Table 1, Table 3 and Table 4). GFLV RNA2-encoded proteins provided much less confident predictions for which only the biological processes provided new insights; protein 2A^HP/Sd^ was predicted to be involved with the regulation of GTPase activity, intracellular signal transduction, and the positive regulation of hydrolase activity for GFLV strain GHu (Table 3 and Table 4, Figure 7 and Table S4). Predictions for protein 2B^MP^ were suggestive of transition metal ion binding and involvement with metabolic processes; however, across both strains, there were levels of incompleteness or uncertainty (Table 3 and Table 4). Finally, satRNA encoding 3A^?^ provided some new predicted functions, such as metal ion binding or metabolite binding (Table 3, Figure 8 and Table S4), which may be involved in the host molecular process [111].

The above predictions of seemingly unrelated protein functions for an RNA virus are intriguing. Indeed, the T3/T7-like RNA polymerase domain has not been described in related systems. However, it is possible that a conserved structural or sequence pattern has alternative functions or purposes. Likewise, no function, motif, or domain has been described for the elusive satRNA encoding protein. Simply because we do not understand these biological networks does not mean that we should discount the possibilities of their utility to promote virus-host compatibility.

GFLV protein 1D^Pro^ is a 3C-like protease that cleaves in *cis* and *trans* the polyproteins encoded by RNA1 and RNA2, respectively [5,15,16,18,22,94,95,96,97]. Site-directed mutagenesis, by analogy to other known proteases, revealed the crucial three residue active sites required for proteolytic activity, His-Glu-Leu [88]. We considered these residues via in silico predictions and demonstrated additional confidence behind the functional and structural aspects of protein 1D^Pro^ (Figure 5). The 3C-like proteinase of GFLV has been eloquently examined against other nepovirus proteinases through AlphaFold in silico predictions, highlighting the evolutionary constraints on active site configuration [112]. While sequence alignment helps bolster these claims [113], the tertiary models provide additional context to enzymatic activity and relate more to protein function [112].

Additionally, we considered the model for GFLV 1E^Pol*/Sd^ to analyze both functions of putative RdRP and documented symptom determinants. While mutagenesis of lysine to glycine at aa residue 802 in GFLV strain GHu abolishes symptoms in *N. benthamiana*, there are still unknowns behind the mechanism of symptom expression by this residue [20,98]. The model generated in this study is consistent with interactions of protein 1E^Pol*/Sd^ with host protein components, given that residue 802 of 1E^Pol*/Sd^ is exposed on the exterior of the protein at the apex of an alpha helix and not buried in a hydrophobic pocket (Figure 6, arrow). No novel functions were detected at this residue, perhaps due to a lack of homologous templates or folds. Additional experimental work is needed to understand the functional and biological aspects of GFLV protein 1E^Pol*/Sd^.

One of the most elusive GFLV proteins is the satRNA encoding protein 3A^?^ [31,32,33,34,35]. At least two new predicted functions were revealed through the comparison of multiple protein prediction programs, but with low confidence, especially for tertiary structure-based programs (Figure 8 and Table S4). GFLV satRNA molecules are divided into two phylogenetic clades, which have been related to geographical differences and evolutionary divergence [35]. Protein 3A^?^ of these two clades gave rise to two different structural and functional predictions. Clade I satRNAs contained only one consistent domain of a DNA topoisomerase at the N-terminal end, while clade II satRNAs contained both a DNA Pol Subunit cdc27 motif at the same area as the DNA topoisomerase of clade I isolate, in addition to a FBXO25 motif at the C-terminal end (Figure 8 and Table S4). Most structures returned low confidence with simple alpha helices in a globular form, with TM-scores consistently below 0.50 (Figure 8). In addition, template-guided or ab initio modeling consistently returned TM scores below 0.50 (Figure 8, File S1 and Table S3), a common threshold for revealing a high amount of randomness in prediction. While ATPbind and BioLIP in D-I-TASSER predicted nucleotide binding and ion binding motifs for GFLV protein 3A^?^, these results must be taken lightly (File S1, Tables S6 and S7). C-I-TASSER, again, did not differ in confidence against homology-guided algorithms when comparing against the available structures in the PDB. The projected accuracy of these TM scores could, in fact, reflect that satellite RNA molecules associated with RNA viruses remain elusive in terms of function and structure.

Our study revealed multiple predicted nucleotide binding sites (Tables S6 and S7), transmembrane domains/helices or pore-lining domains (Table S8), and subcellular localization of GFLV proteins and satRNA protein (Table 3, Table 4, Tables S4 and S5). While NsitePred [84] (Table S6) relies on sequence-based detection of nucleotide binding sites, ATPbind [83] (Table S7) relies on structure-based detection. This may explain the outcome of varying numbers and residues of nucleotide binding sites detected between these two programs (Tables S6 and S7). GFLV protein 1B^Hel*/VSR^ contained the nucleotide binding sites with the highest probabilities (0.80–1.0), which were observed in both programs (Tables S6 and S7). Numerous differences in predicted ATP binding site counts for proteins 1A^VSR^ (difference of 10 sites) and 2A^HP/Sd^ (difference of 9 sites) were observed between GFLV strains F13 and GHu (Table S7). These findings highlight the importance of incorporating sequence and structure homology into the prediction models when characterizing proteins in silico. Unlike nucleotide binding site prediction, a total of nine transmembrane domains/helices (only considering those that were detected from at least two out of the seven prediction programs) were present in three GFLV proteins (three in 1B^Hel*/VSR^ of GFLV strains F13 and GHu; one in 1E^Pol*/Sd^ of GFLV strain GHu; one in 2C^CP/Td^ of GFLV strains F13 and GHu) (Table S8). Phyre2 predicted the transmembrane domain in protein 1E^Pol*/Sd^ of GFLV strain GHu but not strain F13. Knowing the composition of the transmembrane domain provides information regarding possible subcellular localization, as well as putative functions, which include but are not limited to membrane transporting, cell-to-cell signaling, and energy transfer [114,115]. For the subcellular localization prediction, most of the GFLV proteins were predicted to be localized in the nucleus (1A^VSR^), cytoplasm (1D^Pro^, 1E^Pol*/Sd^, and 2B^MP^), or mitochondrion (2A^HP/Sd^), while most satRNA-encoded proteins were estimated to be localized in either the plastid or nucleus (Table 3, Table 4 and Table S5). Protein 1B^Hel?*/VSR^ was predicted to be localized in host intracellular membrane-bounded organelle such as endoplasmic reticulum, confirming previous findings [102] (Table 1, Table 3, Table 4 and Table S5). Some of these predictions also confirmed previous findings of protein 2B^MP^ localizing in the plasmodesmata [99,100] (Table 1, Table 3 and Table 4). Subcellular localization experiments via microscopy would be useful to confirm those predictions and better understand interactions among GFLV proteins and host proteins.

Viral proteins utilize various capacities to perform multiple functions, often linked to binding pockets for a suitable substrate. With the release of AlphaFold2, there has been a resurgence in investigating molecular inhibitors of proteins related to disease and undesirable phenotypes [116,117]. Furthermore, cryptic pockets are unexposed and difficult to study, but further structural understandings can unravel additional mechanisms of enzyme or functional inhibition [118]. The more data collected and experimentally resolved, the better we can understand the molecular mechanisms of virus-specific protein functions. By focusing on these binding pockets, one may identify potential ways to inhibit protein function through the discovery of allosteric sites or altered protein structures that depend on the opening or closing of the pockets [119]. Predictions on binding pockets and protein interactions were made for at least every D-I-TASSER prediction. For example, the proteolysis His-Glu-Leu pocket of GFLV protein 1D^Pro^ and the different core RdRP motifs of GFLV protein 1E^Pol/Sd^ were modeled with high confidence (Figure 6).

Other studies have leveraged AlphaFold2 predictions to focus on resolving structural components via crystallography [120,121]. We incurred an overabundance of computational software to analyze proteins encoded by GFLV and sought to define the best models. Using 28 independent programs (Table 2 and Figure 2), we were able to construct structure models of every GFLV protein, predict functional characteristics, and compare these against functions reported in the literature (Table 1, Table 3 and Table 4). While we confirmed some functional characteristics of GFLV proteins, we also uncovered potential undescribed functions. Homology-guided and ab initio protein prediction programs are difficult to compare when GFLV proteins have varied levels of understanding. Nevertheless, we utilized standards of the field to effectively compare the output of each program’s prediction and weigh each accordingly. Each program used for predictive modeling brought individual strengths and weaknesses toward our goal of annotating the GFLV proteome (Figure 2). MotifFinder (GenomeNet) and Phyre2 [79] relied heavily on sequence alignment and secondary structure to construct a piece-wise understanding of protein products. These programs were fast and provided easy manipulation of search parameters (reducing stringencies of similarity or providing short sequences that were more similar) (Table S3). Programs used to generate three-dimensional protein structures ab initio (AlphaFold2, D-I-TASSER, and MTD-TASSER) were more computationally intensive and took more time to yield outputs if using open-source servers [41,42,43,44,45,46,47,49,50]. These programs require more RAM and storage on local systems, which can prevent high throughput analysis. The use of D-I-TASSER [44] often provided the most confident results (Figure 3 and Table S3). Six GFLV protein sequences returned with the highest TM scores through the I-TASSER suite and averaged 0.57 (Table S3). Although conducting 3D structure-based functional annotation using extended parameters of the D-I-TASSER suite is more time-consuming compared to structural predictions only, it provided the most biologically relevant information about how a protein behaves in vitro in our study.

In silico predictions of protein structures and functions are constrained by the availability and knowledge of the related solved protein structures [122]. Unique sequences or sequences with little similarity to solved structures, like GFLV proteins, are often most challenging for in silico predictions; however, this is intrinsic to the scoring algorithm using known sequences to adjust the probability of certain folds due to protein folding patterns. This topic has been raised in several recent publications [123,124,125]. Our in silico modeling approach showed that prediction algorithms were consistent with previously reported findings of the GFLV protein functions and localizations [14,15,16,18,19,36,99,100,102]. The GFLV proteins with known homologous structures crystalized, such as the protease, RdRP, and CP, and returned high confidence values in silico prediction (Figure 3 and Table S3). As expected, proteins with little to no homology, unique functions, or lacking within the PDB returned low confidence values with homology-based programs (Figure 3 and Table S3). GFLV proteins such as the VSRs, putative helicase 1B^Hel*/VSR^, 1C^VPg^, homing protein 2A^HP/Sd^, and movement protein 2B^MP^ all benefitted from some level of refinement or ab initio programs, performing even higher than homology-guided algorithms (Figure 3 and Table S3).

In conclusion, we leveraged the value and power of in silico prediction and characterization of virus proteins to better understand the multifunctionality and structure of GFLV proteins and gain new insights into their potential roles in virus pathogenicity. The use of a diverse pool of predictive models and programs to study GFLV proteins enabled us to evaluate the advantages, limitations, and performance of each program. GFLV strain-specific predictions and predictions common to GFLV strains F13 and GHu were generated using amino acid sequences of the two virus strains. After such in silico analyses, we retain that a central and uniformly controlled calculation for model confidence should be employed by protein prediction algorithms for accurate comparisons. Validating the models through, for instance, biological assays or X-ray crystallography following protein purification and crystal production will be essential in ensuring their accuracy and reliability.

## Figures and Tables

**Table 2:** List of protein prediction algorithms utilized to investigate the grapevine fanleaf virus (GFLV) protein repertoire.

	Program	Algorithm	Modeling Method	Output	Confidence Metric	Confidence Range	Reference
1	AlphaFold2 (ColabFold)	Neural network	Template based	Tertiary protein model	eTM and pLDDT	0.00–1.00 and 0.00–100	[41,42,43]
2	C-Quark	Contact-assisted *ab initio*	Free modeling	Tertiary protein model	eTM	0.00–1.00	[61]
3	C-I-TASSER	Neural network	Free modeling	Tertiary protein model	pTM/TM-align	0.00–1.00	[48,51]
4	D-I-TASSER	Neural network	Template based	Tertiary protein model	pTM/TM-align	0.00–1.00	[44,45,46,47,48]
5	D-I-TASSER MTD	Neural network	Template based	Tertiary protein model	pTM/TM-align	0.00–1.00	[48,49,50]
6	ESMFold	Neural network	Single input sequence	Tertiary protein model	pTM/pLDDT	0.00–1.00 and 0.00–100	[56]
7	Robetta	Extension of RoseTTAFold and trRosetta	Free modeling	Tertiary protein model	GDT	0.00–1.00	[62,63,64]
8	trRosetta	Neural network	Free modeling	Tertiary protein model	eTM/pLDDT	0.00–1.00 and 0.00–100	[59,60]
9	BioLiP	Semi-manually curated database	Guided	Functional predictions	C-Score	0.00–1.00	[48]
10	RGN2	Neural network	Free modeling	Tertiary protein model	GDT	0.00–1.00	[57]
11	ProtGPT2	Neural network	Free modeling	Tertiary protein model	pLDDT	0.00–100	[58]
12	QMEANDisCo	Distance based model quality estimation	NA	Confidence metric	QMEANDisCo	0.00–100	[92]
13	Phyre2	Multiple sequence alignment	Multiple sequence alignment	Secondary/tertiary protein model	Coverage and Identity	0.00–100	[79]
14	palmID	Sequence conservation	NA	Confidence for RdRP	RdRP score	0.00–100	[80]
15	MOTIF Search	Sequence conservation	Multiple sequence alignment	Primary sequence domain detection	E-value or *p*-value	Lower *p*-value is better	[69,70,71,72]
16	CATH/Gene3D	Semi-manually curated database	Multiple sequence alignment	Primary sequence domain detection	Extensive metrics	Multiple	[81,82]
17	LOCALIZER	Machine learning	Feature detection based on known sequences	Primary sequence domain detection	Ranking	Priority ranking	[73]
18	Plant mSubP	Machine learning	Feature detection based on known sequences	Primary sequence domain detection	Percentage probability	0.00–1.00 (Percentage)	[74]
19	MultiLoc2	Machine learning	Feature detection based on known sequences	Primary sequence domain detection	Percentage probability	0.00–1.00 (Percentage)	[75]
20	TargetP ^a^	Neural network	Feature detection based on known sequences	Primary sequence domain detection	Ranking	Percentage	[76]
21	SignalP ^a^	Neural network	Feature detection based on known sequences	Primary sequence domain detection	Ranking	Percentage	[77]
22	MembraneFold ^a^	AlphaFold/OmegaFold based	Feature detection based on known sequences	Primary sequence domain detection	pLDDT for membrane proteins	0.00–100	[85]
23	DeepTMHMM ^a^	Neural network	Feature detection based on known sequences	Primary sequence domain detection	Probability	0.00–1.00	[86]
24	Split 4.0	Consensus hidden Markov model	Feature detection based on known sequences	Primary sequence domain detection	Binary	Threshold	[87]
25	Phobius	Hidden Markov model	Feature detection based on known sequences	Primary sequence domain detection	Probability	Threshold	[88]
26	MEMESAT3 (PSIPRED)	Hidden Markov model (HMM) and model recognition	Feature detection based on known sequences	Primary sequence domain detection	Log likelihood ratio	Threshold	[89]
27	ATPbind	Machine learning	Feature detection based on structural configuation	Tertiary sequence domain detection	Identity of residues	Threshold	[83]
28	NsitePred	Machine learning	Feature detection based on known sequences	Primary sequence domain detection	Probability	0.00–1.00	[84]

^a^ None or irrelevant predictions were obtained with these programs for GFLV proteins.

## Data Availability

All data analyzed for the has been compiled into the supplementary information available with the manuscript online. Top models for proteins generated in this work were submitted to ModelArchive under ID numbers: ma-nqabp, ma-gzevc, ma-3h6c, ma-wg6ry, ma-pufyr, ma-gs2nn, and ma-yhs0u. Please contact the corresponding author with any additional requests.

## Supplementary Materials

The following supporting information can be downloaded at: https://www.mdpi.com/article/10.3390/biom14010062/s1, File S1. Protein data bank (.pdb) formatted files for grapevine fanleaf virus proteins generated with programs used in this study; Figure S1. Grapevine fanleaf virus coat protein predictions align well with the already resolved crystalline structure PDB:5FOJ.; Table S1. Accessions of grapevine fanleaf virus used in this study; Table S2. Amino acid sequences of grapevine fanleaf virus curated for use in this study; Table S3. Reporting metrics for protein prediction software in the determination of protein structure; Table S4. Curated functional annotations of grapevine fanleaf virus proteins through D-I-TASSER predictions of protein structure; Table S5. Prediction table of subcellular localization of proteins encoded by grapevine fanleaf virus strains F13 and GHu and satRNAs; Table S6. Nucleotide binding-site prediction table; Table S7. Binding site prediction; Table S8. Prediction table of transmembrane domains/helices and/or pore-lining domains.
