# Supplementary material for: Predictive Modeling of Proteins Encoded by a Plant Virus Sheds a New Light on Their Structure and Inherent Multifunctionality
Source: Biomolecules. 2024 Jan 2;14(1):62. doi: 10.3390/biom14010062 (PMC10813169; doi:10.3390/biom14010062)
Supplement: Supplementary file 1 [file biomolecules-14-00062-s001.zip › biomolecules-2773710-Figure S1 Coat protein 2C CP alignment.pdf]

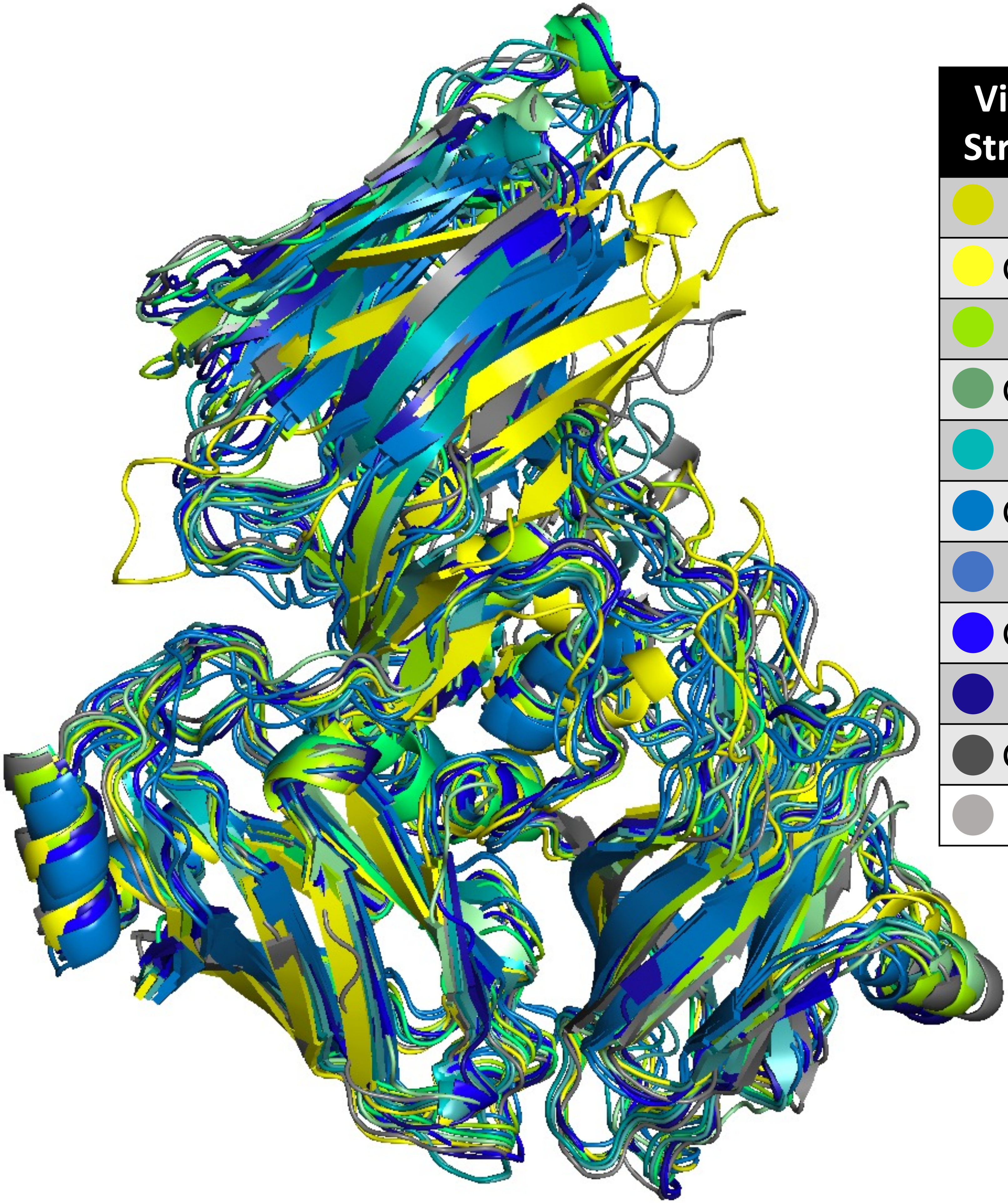

Viral Strain	Prediction Software	RMSD
F13	AlphaFold2	1.810
GHu	AlphaFold2	3.718
F13	D-I-TASSER	1.641
GHu	D-I-TASSER	1.432
F13	ESM	1.825
GHu	ESM	1.862
F13	Robetta	19.773
GHu	Robetta	14.505
F13	trRosetta	1.610
GHu	trRosetta	1.553
**PDB:5FOJ**		
