# Supplementary material for: Predictive Modeling of Proteins Encoded by a Plant Virus Sheds a New Light on Their Structure and Inherent Multifunctionality
Source: Biomolecules. 2024 Jan 2;14(1):62. doi: 10.3390/biom14010062 (PMC10813169; doi:10.3390/biom14010062)
Supplement: Supplementary file 1 [file biomolecules-14-00062-s001.zip › biomolecules-2773710-Supplementary_materials_captions.pdf]

Supplementary materials for

Predictive modeling of proteins encoded by a plant virus sheds a new light on their structure and inherent multifunctionality

Brandon G. Roy[1,*], Jiyeong Choi[1], and Marc F. Fuchs[1]

[1]Plant Pathology and Plant-Microbe Biology Section, School of Integrative Plant Science, Cornell University, 15 Castle Creek Drive, Geneva, NY 14456, USA

***Correspondence: bgr36@cornell.edu**

Supplementary Figure S1. Protein data bank (.pdb) formatted files for grapevine fanleaf virus proteins generated with Al-phaFold2, C-I-TASSER, D-I-TASSER (MTD), ESMFold, Robetta, and trRosetta. Associated metadata and accompanying data is presented in the same form as received from each program. (zip)

Supplementary Table S1. Accessions of grapevine fanleaf virus used in this study.

GFLV RNA molecule	Strain	NCBI Accession
RNA1	F13	NP619689.1
RNA1	GHu	AFM91094.1
RNA2	F13	NC003623.1
RNA2	GHu	ABR09917.1
RNA3	CO2	MH383244.1
RNA3	F13	NC003203.1
RNA3	LR 4/29	ALA99080.1
RNA3	Py17	UZF66112.1
RNA3	REF KE1	ALA99135.1
RNA3	SACH44	AGT42202.1
RNA3	SWT6	KX034963.1
RNA3	ZUP 1/4 c2	ALA99154.1

Supplementary Table S2. Amino acid sequences curated for use in this study pertaining to proteins encoded by grapevine fanleaf virus and the associated strain and NCBI available accessions. (.xlsx)

Supplementary Table S3. Reporting metrics for protein prediction softwares in the determination of protein structure. Programs include AlphaFold2, D-I-TASSER, trRosetta, Robetta, ESMFold, as well as the sequence specific Phyre2 server. Associated metrics of predicted/estimated template modeling (TM) score (pTM/eTM), TM-align scores to known PDB structures, predicted global distance test (GDT), predicted local distance difference test

(pLDDT), alignment coverage, and percent confidence were extracted from such predictions for comparative analysis. *Programs employed only if unfit for standard protein prediction tools. *Yellow highlighted cells represent the highest confidence prediction. (.xlsx)

Supplementary Table S4. Curated functional annotations of grapevine fanleaf virus (GFLV) proteins through D-I-TASSER predictions of protein structure displayed alongside sequence dependent prediction software Motif Search (https://www.genome.jp/tools/motif/) and ScanProsite (Expassy, Swiss Insititute of Bioinformatics). Any annotation included by previous literature is included for convenient comparisons. Predicted molecular function (MF), biological functions (BF), and cellular compartment (CC) from COFACTOR (Zhang et al. 2017), a native protein function prediction tool to the D-I-TASSER server. (.xlsx)

Supplementary Table S5. Prediction table of subcellular localization of proteins encoded by grapevine fanleaf virus strain F13 and GHu and satRNAs. Five programs (LOCALIZER 1.0.4, TargetP, SignalP, Plant mSubP, and MultiLoc2) were used for this prediction. Those that had no prediction are shown as "NA". Those that had prediction probability of ≥ 0.7 are highlighted in gray while those with probability of ≥ 0.8 are highlighted in yellow. (.xlsx)

Supplementary Table S6. Nucleotide binding-site prediction table based on NsitePred (Chen et al. 2012). Those that exhibit ligand binding probability of ≥ 0.8 (max = 1.0) are highlighted in yellow. (.xlsx)

Supplementary Table S7. Binding site prediction encoded by grapevine fanleaf virus proteins according to prediction modeling and previously reported findings. Model that gave the highest confidence score for protein structure prediction was chosen for each protein. (.xlsx)

Supplementary Table S8. Prediction table of transmembrane domains/helices and/or pore-lining domains, encoded by proteins of grapevine fanleaf virus strain F13 and GHu and satRNAs. Six programs (MembraneFold, DeepTMHMM, Splot 4.0, Phobius, MEMSTAT3, and Phyre2) were used to predict amino acid residues that are part of the transmembrane domains/helices. Those that show no transmembrane domains/helices and/or pore-lining domains are indicated by "-". The predicted transmembrane domains/helices amino acid residues that overlapped from two or more programs are shown under "Overlap" column. (.xlsx)
